# SpaBalance: Balanced Learning for Efficient Spatial Multi‐Omics Decoding

**DOI:** 10.1002/advs.202512973

**Published:** 2025-10-14

**Authors:** Yingbo Cui, Yong Zhao, Canqun Yang, Tao Tang, Xiangke Liao, Hongyu Zhang, Huiying Zhao, Zheng Wang, Yuansong Zeng

**Affiliations:** ^1^ College of Computer Science and Technology National University of Defense Technology Changsha 410073 China; ^2^ School of Big Data and Software Engineering Chongqing University Chongqing 401331 China; ^3^ Department of Pathology Department of Medical Research Center Sun Yat‐Sen Memorial Hospital Sun Yat‐Sen University Guangzhou 510120 China; ^4^ Jinfeng Laboratory Chongqing 400039 China; ^5^ School of Computer Science and Engineering Sun Yat‐sen University Guangzhou 510006 China

**Keywords:** cross‐omics integration, multi‐omics balanced learning, private and shared learning, spatial multi‐omics

## Abstract

Recent breakthroughs in spatially resolved multi‐omics have unlocked the ability to simultaneously profile multiple molecular layers within tissues, offering unprecedented insights into their coordinated roles in development and disease. Despite these advancements, integrative analysis of multi‐omics data remains a formidable challenge due to inherent biological and technical discrepancies across assays, often leading to gradient conflicts during joint learning. These conflicts arise as optimization trajectories from different omics compete or contradict, thereby constraining integration performance. To overcome this challenge, SpaBalance, a unified computational framework designed to harmonize cross‐omics learning via gradient coordination and adaptive feature decomposition, is proposed. SpaBalance introduces a novel gradient equilibrium mechanism that dynamically balances inter‐omics contributions during backpropagation, resolving conflicts through task‐specific prioritization without requiring manual weighting. Concurrently, SpaBalance leverages a dual‐stream architecture to simultaneously learn shared representations and preserve omics‐specific features. Extensive evaluations across a variety of spatial omics datasets, including paired epigenome‐transcriptome and proteome‐transcriptome data from human tumors and brain tissues, demonstrate SpaBalance's superior ability to delineate complex spatial domains and uncover previously hidden multi‐omics regulatory hubs, significantly improving clustering accuracy and biological interpretability. Moreover, SpaBalance flexibly scales to integrate multiple omics, bridging data integration with biological discovery and advancing spatially resolved systems biology.

## Graphical abstract

SpaBalance is a computational framework that harmonizes multi‐omics learning via gradient equilibrium and dual‐stream feature decomposition, achieving superior clustering accuracy, biological interpretability, and scalable integration of three or more spatial omics modalities.

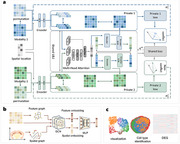

## 1 Introduction

In recent years, spatial multi‐omics technologies have advanced rapidly, enabling the simultaneous acquisition of multi‐layered omics data along with their spatial localization. This breakthrough overcomes the limitations of conventional sequencing methods,^[^ ^1^, ^2^, ^3^, ^4^ ^]^ which rely on aligning sequencing reads to reconstruct transcript structures and measure gene expression levels, but typically lack spatial information. In contrast, spatial multi‐omics integrates spatial localization with molecular profiling, allowing for the spatial mapping of molecular features within tissues. This includes not only spatial transcriptomics,^[^ ^5^, ^6^, ^7^ ^]^ but also extends to spatial epigenomics^[^ ^8^, ^9^ ^]^ and spatial proteomics.^[^ ^10^, ^11^, ^12^ ^]^ These technologies can simultaneously resolve genomic structures, molecular states, and the spatial distribution of cells, providing deep insights into gene regulatory mechanisms and the complex relationships within the tissue microenvironment. Among them, spatially resolved transcriptomics, also known as spatial transcriptomics, was recognized as the “Method of the Year” by Nature in 2020.^[^ ^13^ ^]^ As a key subfield, it preserves tissue structural information while resolving the spatial distribution of transcripts. Currently, spatial multi‐omics has gained widespread attention and was further highlighted by Nature in 2022 as one of the seven key technologies.^[^ ^14^ ^]^ This approach not only integrates transcriptomics, proteomics, and chromatin accessibility data but also captures spatial heterogeneity in cellular functions and microenvironment‐dependent regulatory mechanisms at single‐cell or even subcellular resolution. Recently, commercial imaging‐based spatial omics platforms capable of achieving single‐cell resolution have been continuously developed. Examples include NanoString CosMx (smFISH‐based),^[^ ^15^ ^]^ Vizgen MASCOPE (MERFISH‐based),^[^ ^16^ ^]^ and 10x Genomics Xechium (ISS‐based),^[^ ^17^ ^]^ which have significantly enhanced the field's capacity for high‐resolution spatial analysis. The core objective of spatial multi‐omics is to integrate heterogeneous omics data while preserving spatial tissue structure to accurately characterize cellular spatial identities and deeply analyze gene regulatory networks, cell differentiation trajectories, and cell‐cell interactions. Notably, the incorporation of spatial information enables researchers to identify microenvironment‐dependent molecular mechanisms and explore biological features across different organizational scales. However, due to significant differences in characteristics and distributions across different omics layers, accurately and efficiently integrating spatial multi‐omics data remains a major research challenge.

The integration of spatial multi‐omics data currently faces numerous challenges, primarily arising from significant differences in the number of features between different omics (e.g., the mismatch between the number of measured proteins and transcripts) and their distinct statistical distributions. This challenge becomes even more complex when spatial information is combined with the features of each omics. Existing multi‐modal integration methods can be categorized into three main types: non‐spatial multi‐omics data integration, spatial single‐omics data integration, and spatial multi‐omics data integration. Non‐spatial multi‐omics data integration methods include Seurat WNN,^[^ ^18^ ^]^ MOFA+,^[^ ^19^ ^]^ totalVI,^[^ ^20^ ^]^ and MultiVI.^[^ ^21^ ^]^ Among them, totalVI is specifically designed for RNA and protein omics in CITE‐seq data. It integrates RNA and protein data into a shared low‐dimensional latent space using a variational autoencoder (VAE) framework, effectively accounting for technical biases such as batch effects and protein background noise. This approach enables unbiased cross‐dataset integration and imputes missing protein measurements. Seurat WNN extends omics support through the weighted nearest neighbor (WNN) algorithm, which dynamically adjusts weights based on data similarity. MOFA+ is based on a factor analysis framework and provides a flexible approach for integrating cross‐omics data. MultiVI combines a modular encoder‐decoder architecture with adversarial training to jointly model RNA, ATAC, and protein data, expanding the range of omics that can be integrated. Although these methods effectively integrate multi‐omics data, they do not leverage spatial information, making it difficult to accurately resolve spatially specific biological structures. As a result, they have significant limitations in capturing spatial heterogeneity and identifying spatial domains.

To solve these challenges, spatial single‐omics data integration tools, such as STAGATE,^[^ ^22^ ^]^ GAAEST,^[^ ^23^ ^]^ SpaGCN,^[^ ^24^ ^]^ GraphST,^[^ ^25^ ^]^ and iSpatial,^[^ ^26^ ^]^ combine spatial information with single‐omics modalities, offering unique advantages in analyzing spatial tissue structures. STAGATE employs a graph attention autoencoder to extract spatial dependency features, while GAAEST optimizes spatial embeddings through a three‐level graph attention contrastive learning framework that incorporates local position, global features, and contextual features. GraphST integrates spatial transcriptomic data using cross‐graph self‐supervised contrastive learning, whereas SpaGCN utilizes a Graph Convolutional Network (GCN) to analyze spatial transcriptomic data and supports joint analysis with histological images. Additionally, iSpatial combines scRNA‐seq and spatial transcriptomics (ST) data, leveraging dimensionality reduction, weighted k‐nearest neighbors (KNN), and the Harmony algorithm to infer genome‐wide spatial expression patterns at single‐cell resolution. These methods enhance the ability to decipher spatial structures, providing powerful tools for spatial analysis of single‐omics data.

However, since these tools rely solely on single‐omics data, they fail to fully leverage the complementary information between different omics layers, making it difficult to comprehensively reveal the complex characteristics of cells and the multi‐level biological mechanisms underlying them. As a result, they have significant limitations in analyzing multi‐omics interactions.

With the emergence of an increasing number of spatial multi‐omics technologies capable of characterizing multiple analytes at single‐cell resolution, vertical integration strategies have rapidly evolved by adapting existing non‐spatial single‐cell methods.^[^ ^27^ ^]^ By leveraging spatial information that links cellular states to their respective micro‐ and macro‐environments—such as through graph neural networks—it should be possible to obtain finer‐grained multi‐omics representations of cellular states. State‐of‐the‐art spatial multi‐omics integration methods have made notable advancements. For instance, SpatialGlue^[^ ^28^ ^]^ utilizes a dual‐attention mechanism to enhance the association between spatial and multi‐omics features, while PRAGA^[^ ^29^ ^]^ constructs dynamic graphs to capture latent semantic relationships, enabling deep integration of spatial information with feature semantics. SMODEL,^[^ ^30^ ^]^ an ensemble learning framework based on dual‐graph regularized anchor concept factorization, focuses on spatial domain identification in multi‐omics data, and SpaMICS^[^ ^31^ ^]^ is a deep subspace learning framework that improves spatial domain identification by disentangling consistent and complementary information across multiple omics layers. However, current studies primarily focus on efficiently integrating multi‐omics data but often overlook potential conflicts between multi‐omics and single‐omics learning objectives during model optimization. Such conflicts may lead to imbalanced convergence among omics, limiting the model's ability to understand and interpret multi‐omics data. Despite significant progress, there is still room for improvement. The root cause of this issue is the “omics imbalance” problem, where one dominant omics overshadows others during training, suppressing their contributions and hindering the full utilization of multi‐omics information. The differences in the convergence speeds of different omics lead to inconsistent learning efficiency when the same optimization objective is applied across omics. Moreover, during the integration process, deep multi‐omics models mainly focus on learning shared representations across omics while neglecting omics‐specific features. This can result in the loss of omics‐specific information, thereby compromising the ability to capture key biological signals.

Here, we propose SpaBalance, a balanced deep learning model for spatial multi‐omics efficient integration. SpaBalance dynamically adjusts the learning weights of each omics to ensure the full integration of spatial information and multi‐omics features, achieving precise cross‐omics alignment. Specifically, SpaBalance introduces a gradient conflict coordination mechanism, dynamically adjusting the gradient direction and magnitude of multi‐omics and single‐omics learning objectives. This effectively alleviates the imbalances caused by conflicts between omics, thereby enhancing the integration of spatial multi‐omics data.

Moreover, SpaBalance applies a dual learning strategy combining inter‐omics shared learning and intra‐omics private learning. Inter‐omics shared learning enhances consistency across omics in a common feature space, improving cross‐omics complementarity and strengthening integration. Intra‐omics private learning focuses on preserving unique information within each individual omics, preventing the loss of critical omics‐specific features during integration, and ensuring more accurate biological signal interpretation. Extensive qualitative and quantitative experimental results demonstrate that SpaBalance significantly outperforms existing state‐of‐the‐art methods in aggregating spatial multi‐omics information into structured, interpretable representations, validating its effectiveness and broad applicability. Furthermore, to further verify SpaBalance's effectiveness in balancing multi‐omics learning, we designed and conducted ablation experiments, which revealed that dynamic weight adjustment plays a crucial role in improving model performance, confirming SpaBalance's contribution to spatial multi‐omics data integration.

## 2 Results

### 2.1 Overview of SpaBalance

**Figure 1:**
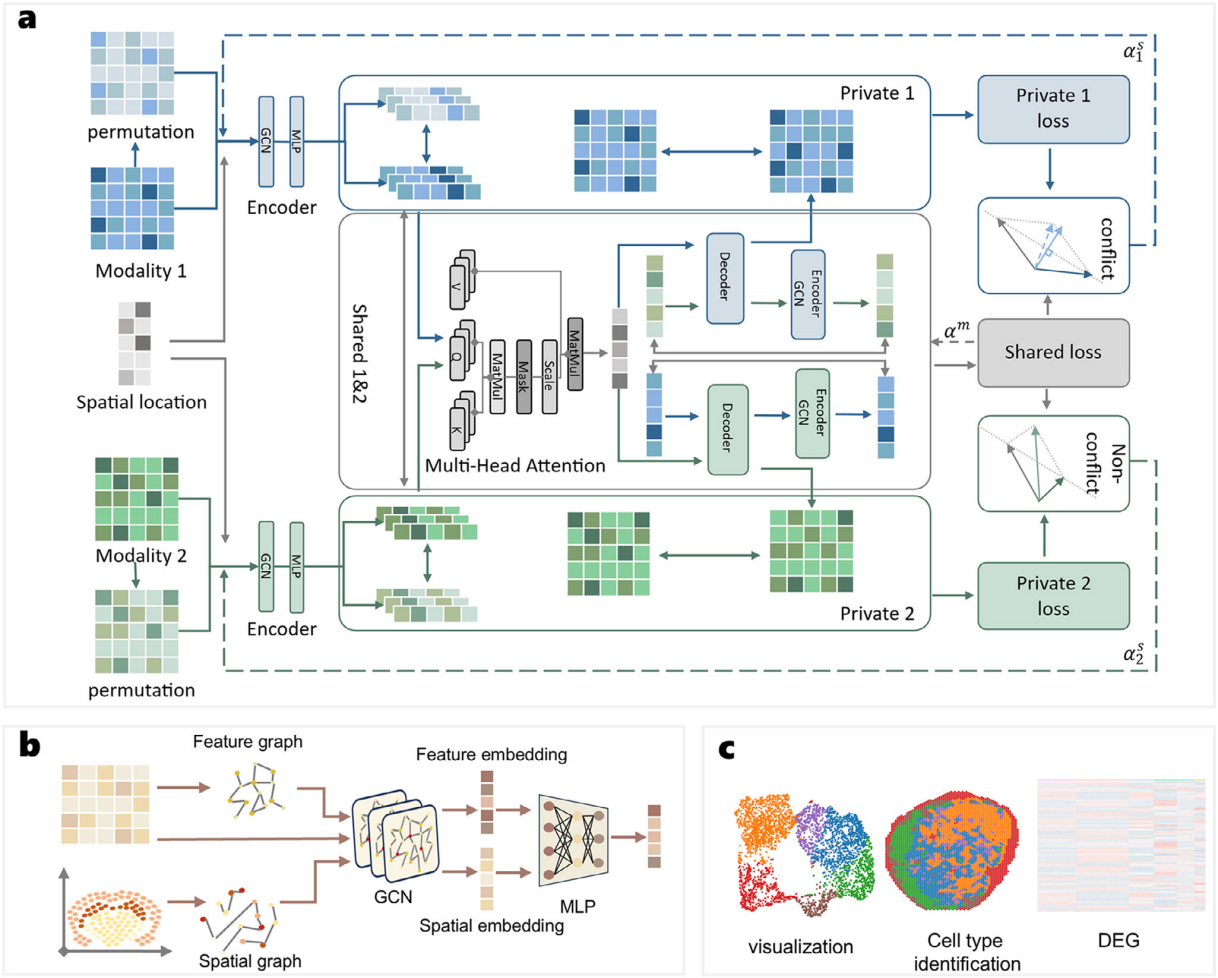
Deep Integration Model for Spatial Multi‐Omics Data Analysis. a) SpaBalance model architecture. To capture interactions between different omics, the SpaBalance learning framework is divided into private learning and shared learning modules. The intra‐omics private learning module extracts omics‐specific representations by integrating graph‐based features. To ensure the importance of each omics is preserved, SpaBalance employs a multi‐head attention mechanism for inter‐omics shared learning, effectively integrating omics‐specific representations to generate the final unified representation. Additionally, to prevent any single omics from dominating the training process, SpaBalance coordinates gradient conflicts to balance learning, ultimately improving the accuracy of the integrated representation. b) Spatial and feature information integration in SpaBalance. To construct a spatial neighborhood graph, SpaBalance first applies a k‐nearest neighbors (KNN) algorithm that incorporates spatial coordinates and normalized expression features from each omics. For each omics, a GNN encoder learns two graph‐specific representations by iteratively aggregating information from neighboring nodes. Subsequently, an MLP module integrates these two graph‐specific representations for each omics, generating a unified single‐omics representation. c) Downstream applications of SpaBalance. By clustering the final integrated representations, we can visualize intra‐cluster cohesion and inter‐cluster separation, identify spatial cellular domains, and conduct differential expression analysis across different spatial regions.

SpaBalance is a spatial multi‐omics balanced learning integration framework designed to balance inter‐omics learning while preserving omics‐specific characteristics during data integration. To achieve this, SpaBalance adopts a dual‐strategy approach: within each omics, it combines graph neural networks (GNNs) and multilayer perceptrons (MLPs) to integrate spatial information with measured features (**Figure** **1**b); across omics, it employs a multi‐head attention mechanism to facilitate cross‐omics information exchange and effective integration. During the integration process, SpaBalance ensures balanced multi‐omics learning by coordinating gradient conflicts, preventing certain omics from dominating training, and mitigating optimization imbalances caused by differences in learning dynamics across omics. To preserve omics‐specific information, SpaBalance implements a dual‐learning strategy consisting of inter‐omics shared learning, which enhances feature consistency and interaction across omics, and intra‐omics private learning, which retains omics‐specific features. This strategy allows SpaBalance to integrate cross‐omics information while maintaining the uniqueness of each omics, resulting in a more comprehensive and biologically meaningful spatial multi‐omics integration (Figure 1a). The integrated representation can further support downstream analyses such as clustering, cell type identification, and differential gene expression analysis (Figure 1c).

Unlike traditional multi‐omics approaches, spatial multi‐omics data carries both molecular features and spatial coordinates for each omics. Due to the inherent spatial heterogeneity of cells, encoding only omics features may fail to fully capture spatial information. To address this, SpaBalance constructs a spatial adjacency graph, embedding preprocessed feature count data into a shared low‐dimensional space. This approach not only preserves the unique semantic information of each omics but also accurately captures cellular spatial heterogeneity. To adaptively learn intra‐omics spatial and feature relationships while ensuring robust information integration, SpaBalance employs a combination of a multi‐layer perceptron (MLP) and a Dropout strategy for intra‐omics integration. The MLP progressively fuses information from different layers, while the Dropout mechanism effectively reduces overfitting risk and enhances model generalization. For inter‐omics integration, simple summation operations may overlook complex relationships and feature disparities between omics, leading to suboptimal integration performance. To overcome this limitation, SpaBalance utilizes a multi‐head attention mechanism, dynamically assigning different weights to each omics. This allows the model to accurately capture relationships and fine‐grained interactions between omics. Multi‐head attention enables parallel learning of inter‐omics correlations across multiple subspaces, resulting in more precise cross‐omics representations and improved delineation of spatial domains or cell types.

However, during model training, uneven omics convergence may cause conflicts between multi‐omics and single‐omics gradients. These conflicts can disrupt the optimization trajectory, leading to insufficient learning for certain omics or even misguiding single‐omics encoders. To mitigate this issue, SpaBalance computes the gradient directions of multi‐omics and single‐omics learning objectives and dynamically adjusts the loss weights between single‐omics and multi‐omics objectives. This ensures that the optimization process effectively balances the learning contributions of each omics, thereby reducing gradient conflicts. Beyond improving the accuracy of shared omics representations, SpaBalance also aims to preserve critical omics‐specific information. To achieve this, we propose a dual‐learning strategy, consisting of inter‐omics shared learning and intra‐omics private learning. Shared learning enhances cross‐omics feature consistency, improving the precision of shared representations, while private learning focuses on omics‐specific features, ensuring that key omics‐specific information is retained during integration.

Ultimately, the integrated multi‐omics representation generated by SpaBalance can be used for biologically relevant spatial domain identification and further clustering analysis to uncover biological features and underlying patterns in spatial multi‐omics data. To evaluate the accuracy of the SpaBalance model, we conducted comparative experiments against other tools on the same datasets. Additionally, we performed a series of ablation studies to validate the effectiveness of multi‐omics balanced learning in SpaBalance.

### 2.2 SpaBalance Performance on Dataset Integrating Three‐Omics

To validate SpaBalance's ability to integrate three‐omics spatial datasets, we tested its performance on a simulated dataset^[^ ^28^ ^]^ containing three omics: RNA, ADT (protein), and ATAC. Before integration, we performed modality‐specific preprocessing for each omics: for RNA, we selected the top 3000 highly variable genes using the Seurat v3 method, normalized the counts per cell, applied log‐transformation, and performed PCA to obtain a feature representation; for ADT, we applied centered log‐ratio (CLR) normalization per cell followed by PCA to generate the feature embedding; for ATAC, we identified 3000 highly variable peaks and performed Latent Semantic Indexing (LSI) to obtain low‐dimensional features.

We initially selected seven representative comparison methods—STAGATE, Seurat WNN, totalVI, MultiVI, MOFA+, SpatialGlue, and PRAGA—which cover spatial single‐omics integration tools, non‐spatial multi‐omics integration tools, and spatial multi‐omics integration tools. This comprehensive benchmarking framework enables a thorough evaluation of SpaBalance in terms of its performance and applicability for cross‐omics data integration. The dataset includes ground‐truth annotations, providing a reliable reference for model evaluation. To comprehensively and objectively assess the integration performance of each method, we employed a variety of supervised evaluation metrics, including Normalized Mutual Information (NMI), Adjusted Mutual Information (AMI), Fowlkes–Mallows Index (FMI), Adjusted Rand Index (ARI), V‐measure, and Completeness. In addition, we utilized an unsupervised Jaccard similarity metric to quantify the intra‐cluster compactness and inter‐cluster separation of the clustering results.

**Figure 2:**
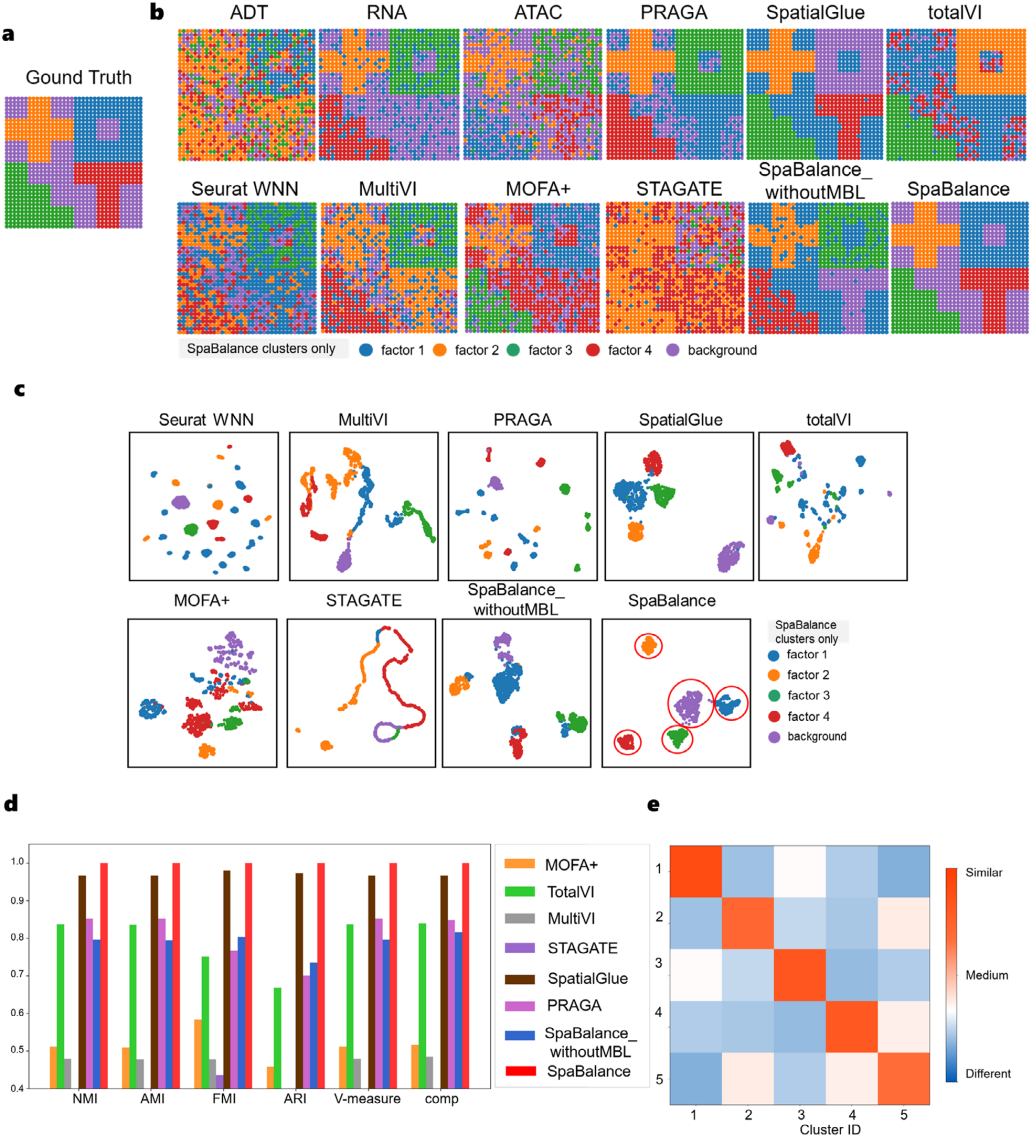
SpaBalance Integrates Three‐Omics Dataset. a) Spatial plot of the ground truth annotations. These annotations were obtained from the original dataset.^[^ ^28^ ^]^ They serve as a reliable reference for evaluating clustering and spatial domain identification. b) Spatial plots of the three‐omics dataset, shown from left to right, the raw data of each individual omics, and the clustering results obtained from single‐cell and spatial multi‐omics integration methods, including STAGATE, Seurat WNN, TotalVI, MultiVI, MOFA+, SpatialGlue, PRAGA, SpaBalance_withoutMBL (multi‐omics balanced learning), and SpaBalance. The annotated labels are based on the SpaBalance results, and the clustering colors may not correspond to the same structures across different methods. c) Clustering results of the integrated embeddings from STAGATE, Seurat WNN, TotalVI, MultiVI, MOFA+, SpatialGlue, PRAGA, SpaBalance_withoutMBL, and SpaBalance are shown. A total of five clusters were identified. While other methods show overlapping clusters, SpaBalance displays clearly separated clusters. The five clusters in the SpaBalance plot are highlighted with red circular borders for clarity. d) Quantitative comparison of MOFA+, TotalVI, MultiVI, STAGATE, SpatialGlue, PRAGA, SpaBalance_withoutMBL, and SpaBalance. Note that Seurat WNN is not shown in this panel because its performance across all quantitative metrics was consistently below 0.4, substantially lower than that of the other methods. e) Heatmap of intra‐cluster compactness and inter‐cluster separability for SpaBalance on the simulated three‐omics dataset, calculated using the unsupervised Jaccard similarity.

The single‐omics visualization results (**Figure** **2**b) show that RNA is primarily associated with factors 1, 2, and 3, while ADT and ATAC provide supplementary information for factors 1, 2, 3, and 4. In terms of spatial factor recovery (Figure 2b), both SpaBalance and SpatialGlue can clearly capture all four spatial factors. However, compared to SpatialGlue, SpaBalance has a better match between the spatial factors and true labels, demonstrating higher factor recognition accuracy. Among the other methods, PRAGA accurately recovers factors 1, 2, and 4, but its recognition of factor 3 remains insufficient. TotalVI performs slightly worse than PRAGA, mainly recovering factors 2 and 3. Seurat WNN, MultiVI, and MOFA+ can capture some factors, but the higher noise levels impact the precise identification of the factors. STAGATE performs the weakest in spatial factor recovery, struggling to accurately restore the true distribution of the spatial factors. SpaBalance demonstrates superior factor recovery capabilities in multi‐omics data integration, providing more precise analytical tools for spatial omics research.

The clustering visualization results (Figure 2c) show that SpaBalance performs the best overall, with compact and clear clustering results, distinct and complete category boundaries, and accurate preservation of spatial structure and omics specificity, demonstrating outstanding multi‐omics integration capability and spatial consistency. Seurat WNN and PRAGA clustering results are sparser, with significant overlap between categories and poor inter‐category separation, failing to accurately capture the complex spatial relationships of multi‐omics data. SpatialGlue clustering results show some inter‐category separation, but intra‐category samples are more dispersed with many outliers, and spatial consistency is not as strong as SpaBalance. STAGATE can capture local spatial topological structures but has limitations in integrating multi‐omics information, with abnormal clustering shapes. It is suitable for single‐omics spatial data but not for multi‐omics integration tasks. MOFA+, MultiVI, and TotalVI clustering results are more dispersed, with blurred category boundaries and significant overlap, failing to effectively integrate multi‐omics and spatial information, resulting in significantly lower integration performance than purpose‐designed spatial multi‐omics frameworks.

In the quantitative evaluation (Figure 2d), compared with Seurat WNN, MOFA+, MultiVI, TotalVI, STAGATE, PRAGA, and SpatialGlue, SpatialGlue achieved the best overall performance, with NMI of 0.965, AMI of 0.965, FMI of 0.979, ARI of 0.973, V‐measure of 0.965, and comp of 0.965. Compared with SpatialGlue, SpaBalance further improved these metrics by 3.5%, 3.498%, 2.1%, 2.7%, 3.499%, and 3.5%, respectively. These results indicate that SpaBalance achieves a representation highly consistent with the ground‐truth distribution and intrinsic features of the simulated dataset. Moreover, the simulated test demonstrates the scalability of SpaBalance, validating its applicability to tasks involving the integration of three or more omics.

In addition, we conducted an ablation study to evaluate the effect of multi‐omics balanced learning (MBL). From both the integration results (Figure 2b,c) and quantitative metrics (Figure 2d), the absence of MBL consistently led to decreased performance. With MBL, SpaBalance exhibited stable convergence across all three omics (RNA, ADT, and ATAC), as reflected in steadily decreasing loss curves (Extended Figure S1a, Supporting Information). The accuracy curves (Extended Figure S1c, Supporting Information) showed continuous improvement of single‐omics and integrated representations throughout training, with the integrated accuracy rapidly increasing and eventually stabilizing near 1.0, suggesting effective cross‐omics alignment and utilization. In contrast, without MBL, the loss curves fluctuated heavily, particularly for ATAC, which remained unstable and high over many epochs (Extended Figure S1b, Supporting Information). Correspondingly, the accuracy curves (Extended Figure S1d, Supporting Information) remained low and unstable, with integrated accuracy failing to improve and persistently staying below 0.6, highlighting an imbalance in multi‐omics integration. These findings demonstrate that MBL is critical for balancing the learning dynamics across omics, preventing dominance of a single omics, and thereby enhancing overall convergence stability and the discriminative power of the integrated representation.

### 2.3 SpaBalance Performance on the Human Lymph Node Dataset

To evaluate the effectiveness of SpaBalance in integrating spatial transcriptomics and proteomics data, and to assess the contribution of its multi‐omics balanced learning mechanism, we conducted systematic benchmarking and ablation studies. These experiments were performed on a real human lymph node dataset,^[^ ^28^ ^]^ generated using the 10x Genomics Visium platform, which captures both RNA and protein omics from the A1 region. This dataset contains ground‐truth biological annotations, providing a reliable reference for model evaluation.

**Figure 3:**
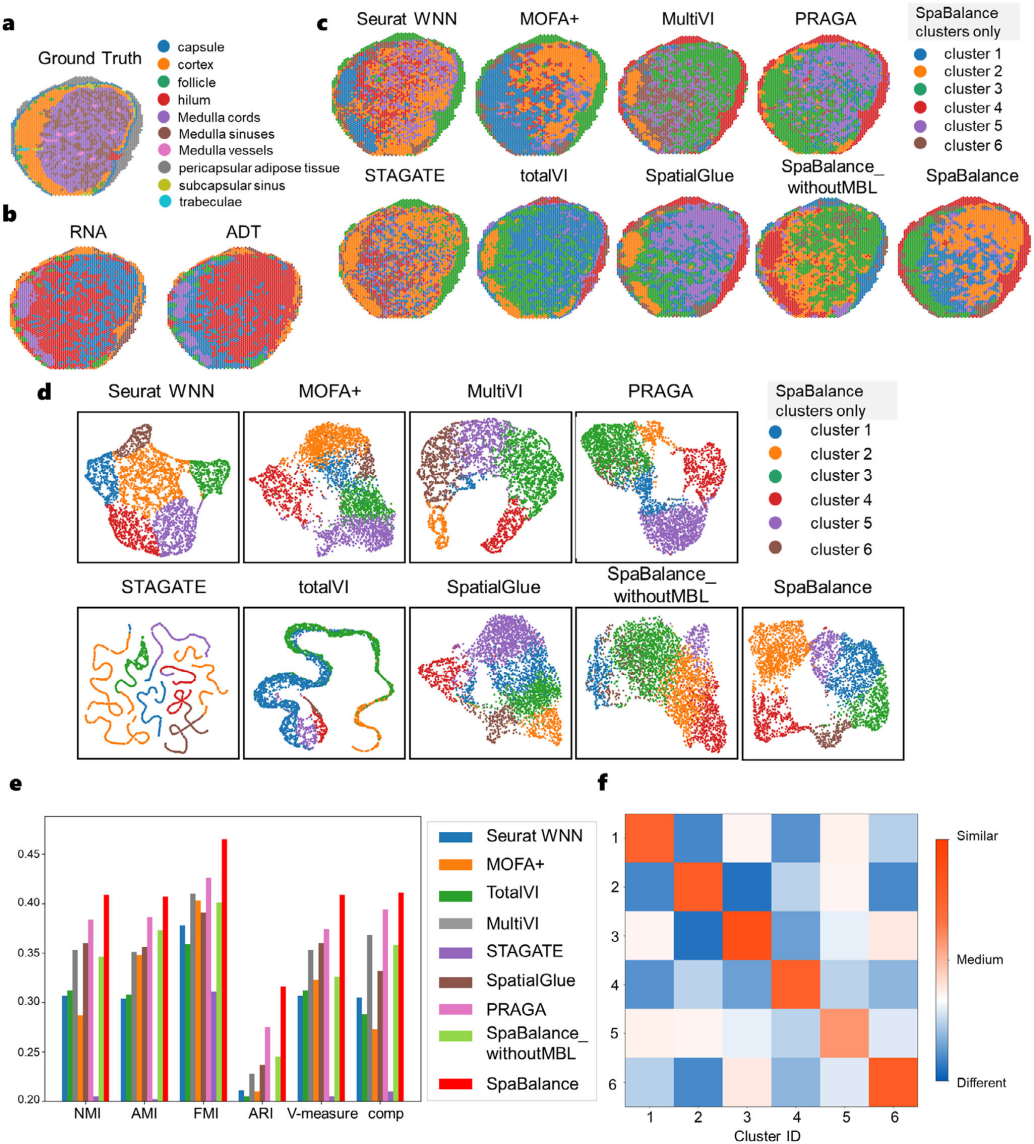
SpaBalance accurately identifies spatial domains in the Human Lymph Node A1. a) Manually annotated human lymph node sample A1, with labels provided by the original dataset^[^ ^28^ ^]^ as reference for evaluating spatial domain identification. b) Spatial plots of single‐cell RNA and protein omics clustering for lymph node sample A1. c) Spatial plots of clustering results from spatial multi‐omics integration methods for lymph node sample A1—STAGATE, Seurat WNN, totalVI, MultiVI, MOFA+, SpatialGlue, PRAGA, SpaBalance_withoutMBL(multi‐omics balanced learning), and SpaBalance. Note that cluster colors do not directly correspond to the same captured structures across different methods. d) Final representation clustering visualization results of STAGATE, Seurat WNN, totalVI, MultiVI, MOFA+, SpatialGlue, PRAGA, SpaBalance_withoutMBL, and SpaBalance. e) Quantitative results of STAGATE, Seurat WNN, totalVI, MultiVI, MOFA+, SpatialGlue, PRAGA, SpaBalance_withoutMBL, and SpaBalance. f) Heatmap of intra‐cluster compactness and inter‐cluster separability for SpaBalance on lymph node sample A1, calculated using the unsupervised Jaccard similarity.

As shown in the visualization of single‐omics features (**Figure** **3**b), both omics are capable of capturing the follicle and parts of the cortex regions, while the RNA omics demonstrates finer resolution by identifying the medullary cords with greater detail. When comparing the integrated embeddings (Figure 3c), although STAGATE exhibits some degree of clustering ability, its clarity and accuracy are relatively limited. In contrast, Seurat WNN, MOFA+, MultiVI, PRAGA, SpatialGlue, and SpaBalance all successfully identify the follicle regions with high accuracy, whereas totalVI fails to do so. In terms of cortex region detection, MOFA+ and PRAGA tend to overestimate the extent, while Seurat WNN, MultiVI, totalVI, and SpatialGlue underestimate its boundaries. Notably, Seurat WNN demonstrates low overall clarity, whereas SpaBalance achieves higher precision in delineating the cortex, highlighting a clear advantage. Furthermore, PRAGA, SpatialGlue, and SpaBalance are all capable of accurately identifying the pericapsular adipose tissue, with SpaBalance showing the most outstanding performance in this aspect. In comparison, PRAGA fails to accurately recognize the medullary cords, and totalVI does not effectively identify the medullary sinuses. Overall, SpaBalance outperforms all other methods in the precision of multi‐region identification, especially in the cortex and pericapsular adipose tissue, demonstrating its superior integrative capability.

Comparing the clustering results of the eight multi‐omics data integration methods (Figure 3d), including Seurat WNN, MOFA+, MultiVI, PRAGA, SpaBalance, STAGATE, totalVI, and SpatialGlue, SpaBalance demonstrates superior performance by maintaining strong separation while flexibly integrating different omics. Unlike other methods, SpaBalance achieves a balanced integration of multi‐omics data, avoiding excessive clustering overlap while preserving inter‐omics details for an optimal integration effect. In contrast, methods such as MOFA+ and MultiVI provide relatively clear cluster separation but lack flexibility in multi‐omics data integration, while PRAGA exhibits more overlap, making it less suitable for tasks requiring distinct clustering boundaries. In addition, we employed an unsupervised Jaccard similarity metric to quantify both intra‐cluster compactness and inter‐cluster separation of the clustering results (Figure 3f). The results indicate strong intra‐cluster cohesion, suggesting consistent grouping within clusters. For inter‐cluster separation, Cluster 1 and Cluster 3, as well as Cluster 3 and Cluster 6, exhibit relatively lower separation scores, likely due to spatial adjacency of cells between these regions. Nevertheless, the separation between other cluster pairs remains highly satisfactory, further confirming the spatial distinctiveness and biological validity of the clustering outcomes.

Using hematoxylin and eosin (H&E) staining‐based annotations as ground truth (Figure 3a), we conducted a quantitative evaluation of the eight integration tools (Figure 3e). Compared to the quantitative results of Seurat WNN, MOFA+, MultiVI, totalVI, STAGATE, PRAGA, and SpatialGlue, PRAGA performed best, achieving the following scores: NMI 0.384, AMI 0.386, FMI 0.426, ARI 0.275, V‐measure 0.347, and comp 0.394. SpaBalance outperformed PRAGA across all metrics, with respective improvements of 2.5%, 2.1%, 3.9%, 4.1%, 3.5%, and 1.7%, further demonstrating its advantage in multi‐omics data integration.

To evaluate the effect of the multi‐omics balanced learning mechanism, we conducted ablation experiments. The results indicate that without balanced multi‐omics learning speeds, the uneven convergence of multi‐omics data during training leads to significant fluctuations in omics loss (Extended Figure S2b, Supporting Information), ultimately reducing model accuracy by 2.4%, 3.4%, 5.4%, 3.7%, 2.3%, and 2.6% across various metrics. Furthermore, analyzing the accuracy trends of single‐omics and integrated representations reveals that without the multi‐omics balanced learning mechanism, overall accuracy declines (Extended Figure S2c, Supporting Information), whereas incorporating this mechanism leads to a clear upward trend (Extended Figure S2d, Supporting Information). These findings strongly indicate that the multi‐omics balanced learning mechanism effectively enhances integration accuracy, ensures balanced optimization across omics, and improves the integration of multi‐omics data.

To ensure that the results are not biased by a specific tissue slice, we further conducted experiments on a real human lymph node dataset^[^ ^28^ ^]^ sampled from the D1 region. As shown in the results (Extended Figure S3a–d, Supporting Information), SpatialGlue, PRAGA, and SpaBalance achieve clearer spatial representations compared to other methods. Notably, PRAGA and SpaBalance provide more accurate localization of the cortex region than SpatialGlue. Furthermore, compared to both SpatialGlue and PRAGA, SpaBalance demonstrates superior capability in accurately identifying the B cell follicle and excluding regions, highlighting its enhanced spatial recognition performance.

### 2.4 SpaBalance Performance on Mouse Brain Coronal Tissue

**Figure 4:**
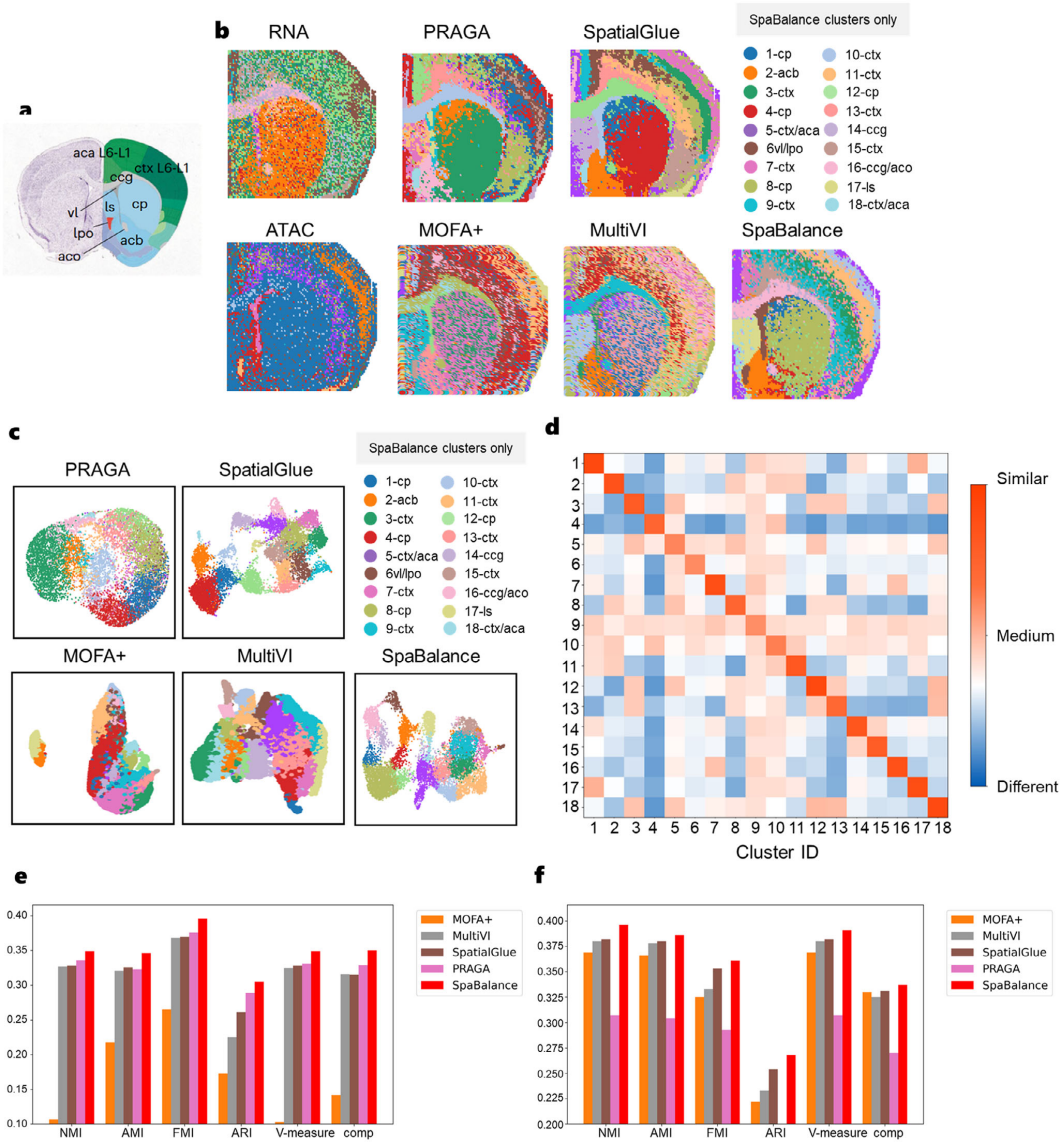
SpaBalance Dissects the Spatial Epigenomic and Transcriptomic Mouse Brain Sample with Higher Resolution. a) Annotation reference of the Allen Mouse Brain Atlas for coronal brain slices of the mouse brain. b) Monopolar clustering (left) and spatial clustering results (right) of single‐cell and spatial multi‐omics integration methods (MultiVI, MOFA+, SpatialGlue, PRAGA, and SpaBalance) using RNA‐seq and ATAC‐seq data. The labeled annotations correspond to SpaBalance results, and clustering colors do not necessarily match the same structures across other methods. ctx, cerebral cortex; cp, caudate putamen; vl, lateral ventricle; lpo, lateral preoptic area; aca, anterior cingulate area; ls, lateral septal nucleus; aco, olfactory commissure; acb, nucleus accumbens; cc, corpus callosum. c) Final representation clustering visualization results of MultiVI, MOFA+, SpatialGlue, PRAGA, and SpaBalance. d) Heatmap of intra‐cluster compactness and inter‐cluster separability for SpaBalance calculated using the unsupervised Jaccard similarity. e) Quantification results of MultiVI, MOFA+, SpatialGlue, PRAGA, and SpaBalance under the ATAC label. f) Quantification results of MultiVI, MOFA+, SpatialGlue, PRAGA, and SpaBalance under the RNA label.

To further validate the performance of SpaBalance and its ability to retain key omics‐specific information during integration, we tested it on a mouse brain coronal section dataset^[^ ^10^ ^]^ from postnatal day 22 (P22). This dataset was generated using spatial ATAC‐RNA sequencing technology, containing 9215 cells and encompassing mRNA and open chromatin region data. Due to the complexity of the data and the higher difficulty in integration, this experiment provides a more challenging test scenario for evaluating the robustness and applicability of SpaBalance in cross‐omics data integration. We used the Allen Brain Atlas (**Figure** **4**a) as a reference to label the following brain regions: cerebral cortex (ctx), caudate putamen (cp), lateral ventricle (vl), lateral preoptic area (lpo), anterior cingulate area (aca), lateral septal nucleus (ls), olfactory commissure (aco), nucleus accumbens (acb), and corpus callosum (ccg).

For benchmarking, since totalVI is only applicable to CITE‐seq data and STAGATE encountered a memory overflow issue under the same experimental environment, we compared MultiVI, MOFA+, SpatialGlue, PRAGA, and SpaBalance. We first visualized the results of individual omics (Figure 4b) and found that they captured various regions with different levels of precision. The results showed that RNA omics data clearly captured the corpus callosum (ccg) and olfactory commissure (aco) regions, while also clearly capturing the caudate putamen (cp) region. The ATAC omics was able to roughly reflect the lateral ventricle (vl) and lateral preoptic area (lpo) regions. Comparing the performance of different tools (Figure 4b), we found that all methods effectively captured the corpus callosum (ccg) and lateral preoptic area (lpo) regions. However, MOFA+ had relatively low clarity in capturing the corpus callosum (ccg) region, and except for PRAGA, the other tools were able to identify the olfactory commissure (aco) region. Overall, MultiVI, and MOFA+ exhibited some limitations in spatial clarity and detail presentation. Among the methods, SpaBalance and SpatialGlue stood out, accurately capturing all key anatomical regions, and after integrating spatial information, outperformed non‐spatial multi‐omics integration tools. Notably, SpaBalance showed a more complete capture of the caudate putamen (cp) region. Although PRAGA could also identify all regions except the olfactory commissure (aco), the clustering plot (Figure 4c) revealed high overlap between omics. Similarly, we quantified the intra‐cluster compactness and inter‐cluster separation of the clustering results (Figure 4d) using an unsupervised Jaccard similarity metric. The results demonstrate that both intra‐cluster and inter‐cluster performance are generally satisfactory, indicating good consistency and separability in capturing spatial structures.

Under the ATAC_clusters label (Figure 4e), compared with MOFA+, MultiVI, TotalVI, PRAGA, and SpatialGlue, PRAGA performed the best in metrics such as NMI, FMI, ARI, V‐measure, and comp, with results of 0.336, 0.376, 0.289, 0.331, and 0.329, respectively. SpatialGlue ranked first in the AMI metric, with a value of 0.326. Compared to these results, SpaBalance improved these metrics by 1.3%, 2%, 2%, 1.6%, 1.8%, and 2.1% for NMI, AMI, FMI, ARI, V‐measure, and comp, respectively, and ranked first overall. Under the RNA_clusters label (Figure 4f), SpatialGlue performed the best among MOFA+, MultiVI, TotalVI, and PRAGA, with quantification results of 0.382, 0.380, 0.353, 0.254, 0.382, and 0.331 for NMI, AMI, FMI, ARI, V‐measure, and comp, respectively. Compared with SpatialGlue, SpaBalance improved by 1.4%, 0.6%, 0.8%, 1.4%, 0.9%, and 0.6% in NMI, AMI, FMI, ARI, V‐measure, and comp, respectively.

**Figure 5:**
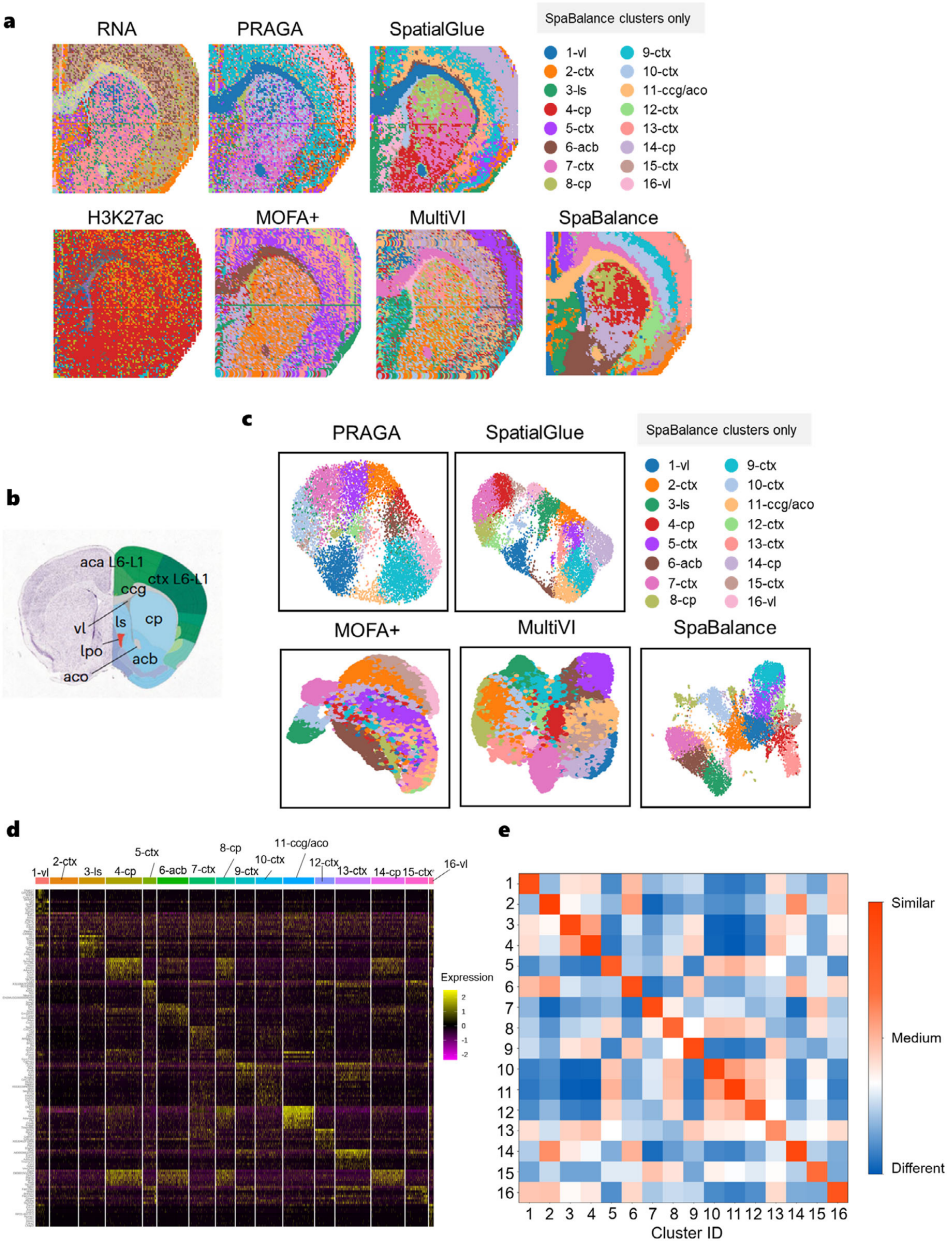
SpaBalance dissects more complex spatial epigenome‐transcriptome mouse brain samples. a) Single‐cell and spatial multi‐omics integration methods' single‐omics clustering (left) and clustering results (right) of RNA‐seq and CUT&Tag‐seq (H3K27ac) data spatial plots –MultiVI, MOFA+, SpatialGlue, PRAGA, and SpaBalance. Annotated labels correspond to SpaBalance's results, and clustering colors do not necessarily match the same structures across other methods. b) Annotation reference of the Allen Mouse Brain Atlas for coronal brain slices of the mouse brain. c) Final representation clustering visualization results for MultiVI, MOFA+, SpatialGlue, PRAGA, and SpaBalance. d) DEG heatmap for each cluster. e, Heatmap of intra‐cluster compactness and inter‐cluster separability for SpaBalance calculated using the unsupervised Jaccard similarity.

We further extended the analysis to another P22 mouse brain dataset,^[^ ^10^ ^]^ which includes coronal sections similar to the previously analyzed mouse brain dataset but contains two omics: RNA‐seq and CUT&Tag. The CUT&Tag data mainly captures the H3K27ac histone modification, which is an acetylation modification at lysine 27 on histone H3. This epigenetic mark is closely associated with active enhancer regions and gene transcription activation. Since this dataset also lacks labeled true tags, we again utilized the Allen Brain Atlas as a spatial reference to annotate and compare the anatomical regions. From the single‐omics images (**Figure** **5**a), it is evident that the RNA data captures the general regions of ccg and aco, while H3K27ac data reflects the vl region. In this dataset, all six tools were able to capture the ccg, aco, and vl regions, but PRAGA, MOFA+, and MultiVI had relatively low overall image clarity. SpaBalance captured the cp (6, 9), acb (13), and ls (1) regions, although SpatialGlue also captured these parts, it struggled to clearly distinguish between acb and cp regions and had more overall noise.

By analyzing the SpaBalance clustering results (Figure 5e) using an unsupervised Jaccard similarity metric, we observed a clear degree of inter‐cluster dissimilarity even among clusters assigned to the same tissue region. This suggests that while these clusters are spatially adjacent, they may exhibit underlying molecular differences. Further investigation of differentially expressed genes (DEGs) within each cluster (Figure 5d) revealed that multiple subclusters within the cerebral cortex (cortex, ctx) region—specifically clusters 2, 5, 7, 9, 10, 12, 13, and 15—show significant differences in gene expression profiles. This finding reveals a marked degree of spatial heterogeneity in the cortex, indicating that it is not a transcriptionally homogeneous region but is instead partitioned into multiple molecularly distinct functional zones. Such spatial heterogeneity provides important molecular insights into region‐specific cortical functions and lays the foundation for further investigation into the regulatory mechanisms underlying cortical functional differentiation.

Notably, in the cp region, clusters 4, 8, and 14 exhibit two distinct modules of highly expressed genes. Module 1 includes Scn4b, Syndig1, Lrrc10b, Nexn, and Dach1. Among these, Scn4b encodes the sodium channel auxiliary subunit 4, which is widely involved in regulating electrophysiological signaling in the nervous system,^[^ ^32^ ^]^ while Syndig1 modulates synaptic AMPA receptor content and contributes to the maturation of excitatory synapses.^[^ ^33^ ^]^ The high expression of this module suggests that the cp region may be enriched in excitatory neuronal networks involved in the regulation of electrical activity. Module 2 comprises Rgs9, D830015G02Rik, Drd1a, Pde1b, Pde7b, and Rarb. Rgs9 encodes a GTPase‐activating protein that accelerates the deactivation of G‐protein signaling pathways, while Drd1a is a classical dopamine D1 receptor involved in neurotransmitter signaling, synaptic structural modulation, and central nervous system development.^[^ ^34^ ^]^ Pde7b is a cAMP‐specific phosphodiesterase with notably high expression in the brain.^[^ ^35^ ^]^ The prominent expression of this module indicates that the cp region may play a key role in dopamine‐related signaling regulation, suggesting the enrichment of dopaminergic neurons involved in motor control and reward pathways.

In the ccg/aco region (cluster 11), we identified a gene module composed of Cldn11, Mal, Mobp, Gsn, Mog, Adamts4, Pllp, Cryab, and Enpp6. Most of these genes are closely associated with myelination and glial cell functions.^[^ ^36^, ^37^, ^38^ ^]^ For example, Mobp is a marker of oligodendrocytes linked to multiple sclerosis, and Enpp6 is a lipid hydrolase involved in glial cell metabolism. The high expression of this module suggests that the ccg/aco region is likely enriched in mature oligodendrocytes, further supporting its critical role in white matter conduction pathways. In the ls region (cluster 3), we observed elevated expression of Zic1, Trpc4, and Dgkg. Zic1 is a well‐established transcription factor involved in neural development, particularly in the formation of forebrain structures;^[^ ^39^ ^]^ Trpc4 belongs to the TRP calcium channel family and plays a role in regulating neuronal excitability;^[^ ^40^ ^]^ Dgkg is involved in phospholipid signaling and is essential for early embryonic neural tube development. These genes suggest that the ls region may be actively engaged in neurodevelopment and the regulation of neuronal excitability. Additionally, in the vl region(cluster 16), the significantly high expression of Top2a marks it as a potential regional marker gene. Top2a encodes DNA topoisomerase II, a critical molecule for cell proliferation and chromatin remodeling. Its high expression suggests the vl region may harbor highly proliferative cells, implying the presence of neural stem cells or progenitor cells concentrated in this area.

### 2.5 SpaBalance Integrates Mouse Thymus and Spleen Datasets

We applied SpaBalance to the mouse thymus slice dataset,^[^ ^12^ ^]^ which was jointly obtained to capture mRNA and protein omics information using the Stereo‐CITE‐seq technology, providing a multidimensional analysis of thymus tissue. The thymus is a key immune organ for T cell development and differentiation, consisting of two lobes, each divided into the central medulla and peripheral cortex layers. The cortex is primarily responsible for the positive selection of immature T cells, while the medulla participates in negative selection to eliminate self‐reactive T cells.

**Figure 6:**
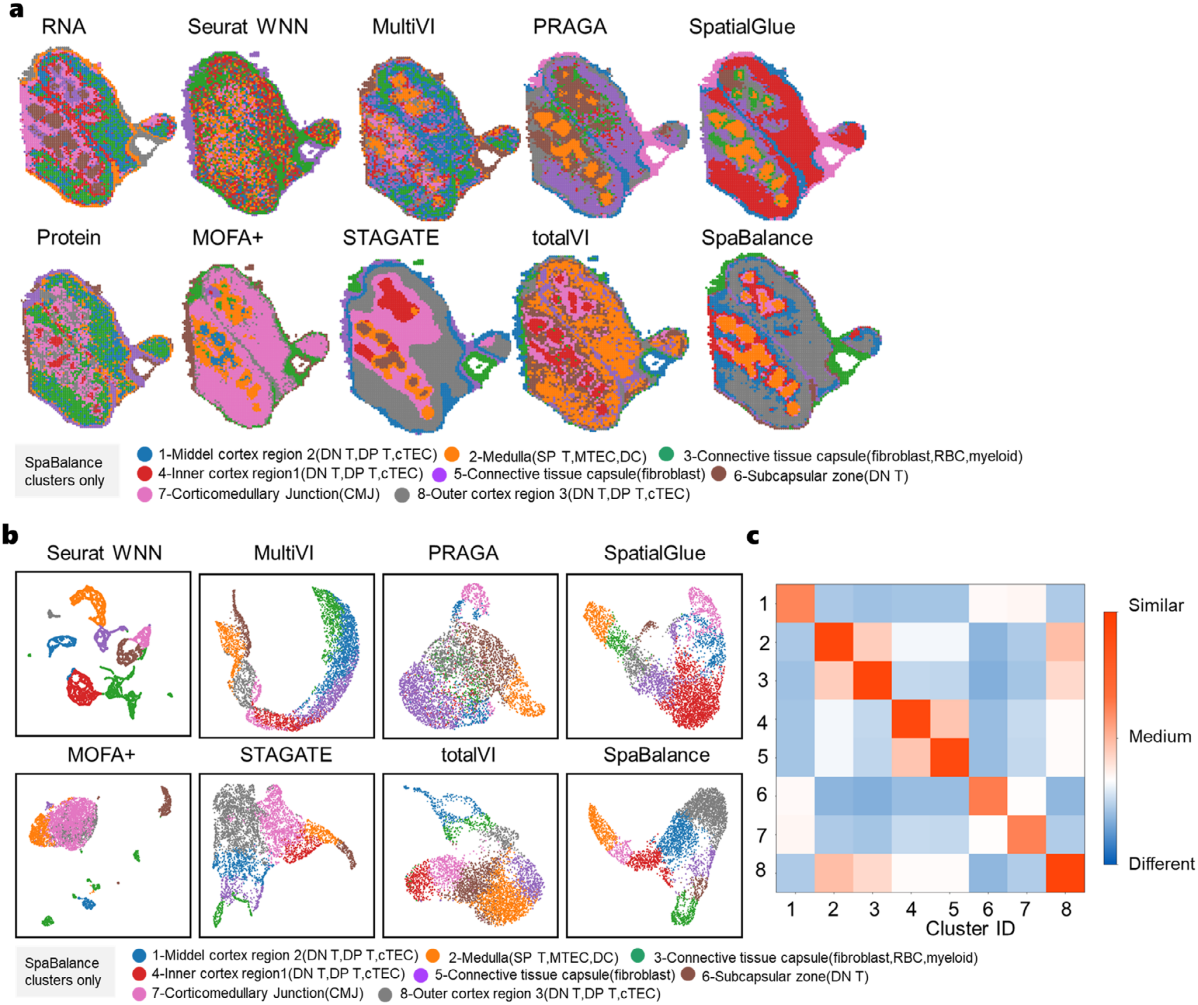
SpaBalance Integrates Mouse Thymus (RNA and Protein Obtained via Stereo‐CITE‐seq). a) Spatial plots of RNA and protein data (mouse thymus, obtained via Stereo‐CITE‐seq) with single‐omics clustering (left), along with a comparison of clustering results from single‐cell and spatial multi‐omics integration methods (right) – STAGATE, Seurat WNN, TotalVI, MultiVI, MOFA+, SpatialGlue, PRAGA, and SpaBalance. Annotated labels correspond to SpaBalance's results, and clustering colors may not match the same structures in other methods. b) Visualization of the integrated embedding clustering results of the mouse thymus for STAGATE, Seurat WNN, TotalVI, MultiVI, MOFA+, SpatialGlue, PRAGA, and SpaBalance. c) Heatmap of intra‐cluster compactness and inter‐cluster separability for SpaBalance on mouse spleen calculated using the unsupervised Jaccard similarity.

By visualizing RNA and protein omics data (**Figure** **6**a), we clearly observe that the outer cortex region and connective tissue capsule (fibroblast, RBC, myeloid) exhibit distinct spatial patterns in both omics, while the RNA omics data further reveals the medulla region. We compared Seurat WNN, TotalVI, MultiVI, MOFA+, STAGATE, SpatialGlue, PRAGA, and SpaBalance and found significant differences in their ability to analyze the thymus spatial structure. MultiVI and STAGATE fail to accurately identify the thymus's bilobed structure, with MultiVI particularly weak in distinguishing the outer cortex region and medulla. Seurat WNN identified parts of the connective tissue capsule, and TotalVI captured the outer cortex region, medulla, and corticomedullary junction, though both methods still showed insufficient spatial resolution. MOFA+, PRAGA, and SpatialGlue performed well in separating the medulla and cortex, identifying the bilobed structure. However, MOFA+ and SpatialGlue did not clearly distinguish the inner cortex region1 and middle cortex region2, and PRAGA showed overlapping regions in the inner cortex region1 and the corticomedullary junction. In contrast, SpaBalance provided superior spatial domain separation, accurately distinguishing the inner cortex and central medulla, presenting clear region delineations and effectively avoiding cell type conintegration due to over‐clustering. Its clustering results (Figure 6b) were stable and refined, capturing local details in complex spatial structures while maintaining the independence of cell populations. The unsupervised Jaccard similarity heatmap (Figure 6c) further demonstrates the model's strong performance in terms of intra‐cluster compactness and inter‐cluster separability.

We also conducted experiments on two additional mouse thymus datasets generated using Stereo‐CITE‐seq. The results (Extended Figure S4a–f, Supporting Information) show that, compared to non‐spatial multi‐omics integration methods, SpatialGlue, PRAGA, and SpaBalance achieved more accurate spatial domain delineation. However, further analysis of the RNA and ADT single‐omics features reveals that SpatialGlue and PRAGA overlooked critical RNA‐derived information in certain regions, leading to less accurate partitioning. In contrast, SpaBalance demonstrated outstanding performance in thymus data interpretation, accurately capturing key anatomical structures and finely distinguishing complex cell types, highlighting its powerful spatial resolution and multi‐omics integration capabilities.

In another experiment, we benchmarked SpaBalance on the spatial analysis dataset^[^ ^11^ ^]^ of the mouse spleen. These data were captured using SPOTS technology, which simultaneously captured transcriptome and extracellular protein data. The spleen is an important organ in the lymphatic system, primarily involved in B‐cell maturation within the germinal centers of B‐cell follicles. These structures are complex and contain various immune cells.

**Figure 7:**
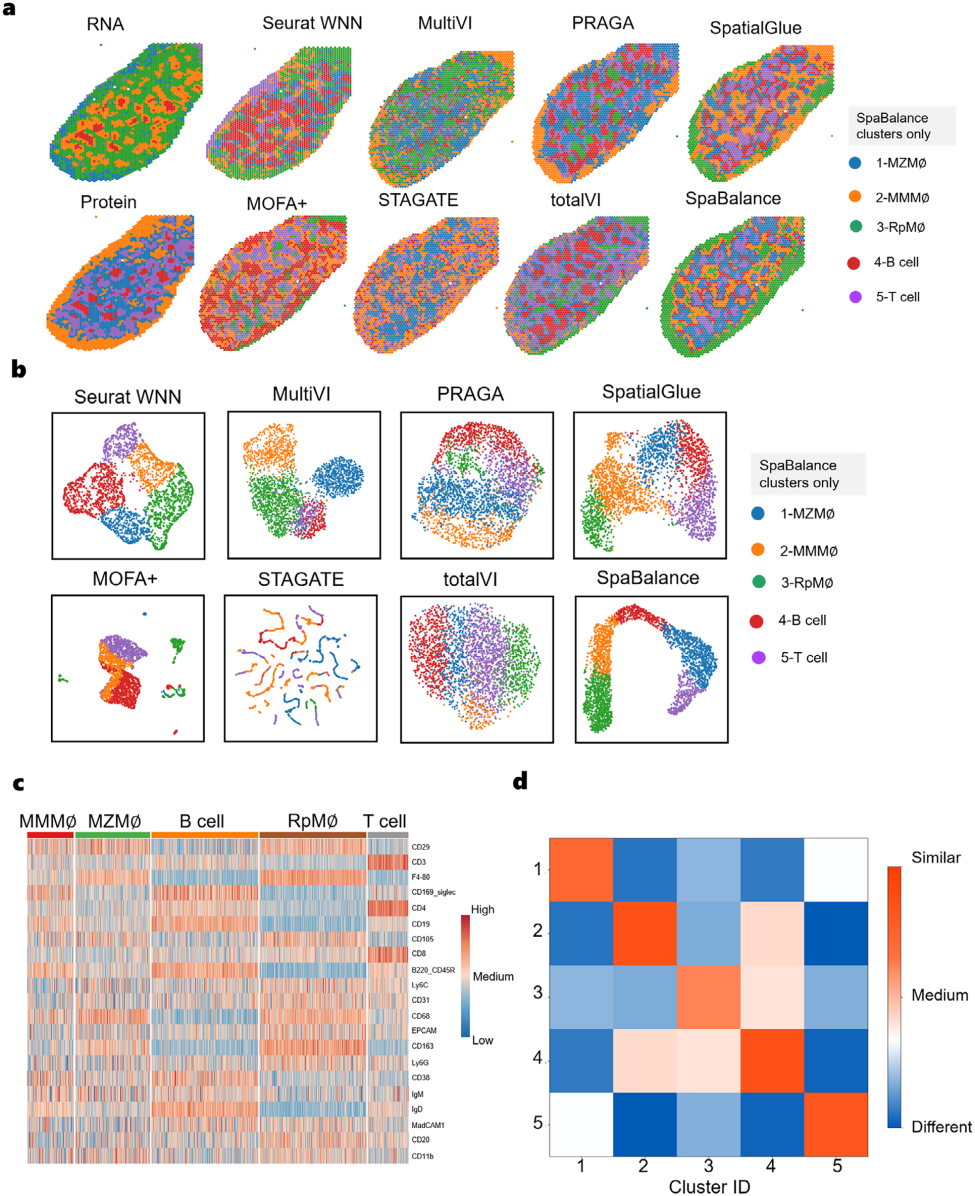
SpaBalance Integrates Mouse Spleen (RNA and Protein Obtained via SPOTS). a) Spatial plots of RNA and protein data (mouse spleen) with single‐omics clustering (left), along with a comparison of clustering results from single‐cell and spatial multi‐omics integration methods (right) – STAGATE, Seurat WNN, TotalVI, MultiVI, MOFA+, SpatialGlue, PRAGA, and SpaBalance. Annotated labels correspond to SpaBalance's results, and clustering colors may not match the same structures in other methods. b) Visualization of the integrated embedding clustering results of the mouse spleen for STAGATE, Seurat WNN, TotalVI, MultiVI, MOFA+, SpatialGlue, PRAGA, and SpaBalance. c) Heatmap of differentially expressed ADTs for each cluster. d) Heatmap of intra‐cluster compactness and inter‐cluster separability for SpaBalance on mouse spleen calculated using the unsupervised Jaccard similarity.

We partitioned the clustering results into five clusters and annotated them, based on their distinct spatial distributions, as MZMΦ, MMMΦ, RpMΦ, B cells, and T cells. From the visualization results (**Figure** **7**a), SpaBalance was able to capture clearer spatial domains. From the clustering results (Figure 7b), MOFA+’s clustering was unclear, with little separation between cell types and significant overlap. STAGATE's clustering was chaotic, particularly in distinguishing cell types, showing substantial overlap between MZMΦ and B cells. While Seurat WNN and MultiVI showed some improvement in clustering, there was still considerable overlap, especially in the separation of RpMΦ, B cells, and T cells. TotalVI showed good performance in separating certain cell types, but the overall clustering was still suboptimal. PRAGA's clustering was more complex, with lower separation between certain cell populations (such as MZMΦ and B cells), resulting in poorer overall clustering performance. SpatialGlue was able to differentiate some cell types well, but the clustering results were not as clear as those of SpaBalance, with some cell populations still showing overlap. SpaBalance, however, clearly distinguished different cell type with high separation between clusters and minimal overlap.

In the ADT marker heatmap (Figure 7c), we first observed that different cell populations exhibit distinct gene expression patterns, clearly distinguishing the major immune cell types. MZMΦ (marginal zone macrophages) and RpMΦ (red pulp macrophages) showed overlap in some highly expressed genes but displayed significant differences in key markers, particularly CD68 and CD163, which were markedly higher in these two populations compared to MMMΦ. Previous studies have demonstrated that CD163 is a highly specific marker of M2‐type tumor‐associated macrophages, primarily expressed on the surface of monocytes and macrophages,^[^ ^41^ ^]^ whereas CD68 is closely associated with the phagocytic activity of macrophages.^[^ ^42^ ^]^ Thus, the heatmap results are consistent with established immunological knowledge and further confirm that CD68 and CD163 may serve as potential molecular markers for distinguishing MMMΦ. Within the B cell population, we detected widespread high expression of CD19 and B220 (PTPRC, CD45R). These classical markers are known to be involved in B cell development, maturation, and activation,^[^ ^43^, ^44^ ^]^ and their spatial distribution in the heatmap verifies the identity of B cells. However, some B cell populations did not exhibit significant gene overexpression, which may reflect functional heterogeneity or low transcriptional activity in a resting state. In addition, CD169 (Siglec‐1) showed prominent expression in certain populations, consistent with its known role in apoptotic cell clearance and antigen presentation,^[^ ^45^ ^]^ suggesting that marginal zone macrophages or B cell–associated subpopulations may contribute to immune regulation. In the T cell population, the prominent expression of CD3, CD4, and CD8 further supported their cellular identity, corresponding respectively to the T cell receptor complex (CD3), helper T cells (CD4), and cytotoxic T cells (CD8).^[^ ^46^, ^47^, ^48^ ^]^ These expression patterns are highly consistent with established immunological features, validating the accuracy of our method in capturing T cell subtypes at the molecular level. Taken together, the results in Figure 7c are not only highly consistent with existing immunological knowledge but also provide clear molecular evidence for distinguishing different immune cell populations. This demonstrates that our method can accurately delineate cell types and offers a reliable foundation for subsequent spatial immunological functional analyses.

These distinct cell types and their marker expression patterns not only showcase the diversity of cells in the immune system but also reflect the specific gene functions in the cells. For example, through differential marker expression, we gain a clearer understanding of each cell type's role in immune responses, inflammation, and tissue repair. The strength of each marker reflects the activity of these genes within the cell, further revealing the functional positioning and contribution of cells in specific immune environments. Through these marker analyses, we can delve deeper into the specific performances and regulatory mechanisms of genes in cell function.

### 2.6 SpaBalance Stability and Robustness to Graph Construction

To evaluate the stability and robustness of SpaBalance, we designed systematic experiments to examine the sensitivity of the method to graph construction parameters. The purpose of these experiments is to determine whether SpaBalance's performance is significantly affected by different choices of the feature neighbor number (k) and spatial neighbor number (r), and to identify robust default settings that can be applied across diverse spatial multi‐omics datasets. We conducted these experiments on both the simulated three‐omics dataset and the human lymph node A1 dataset, quantifying performance.

**Figure 8:**
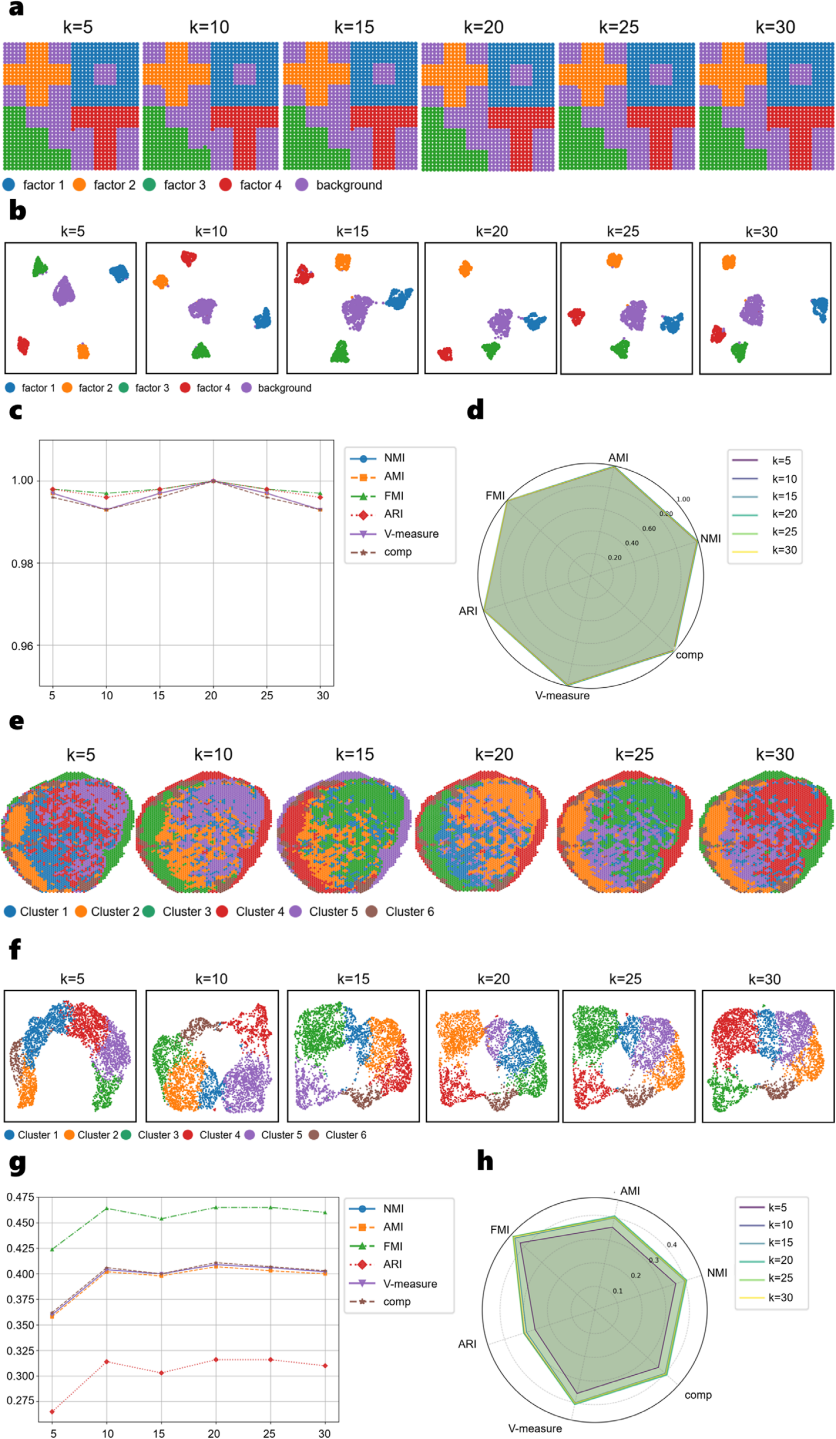
Performance of SpaBalance under Varying Feature Neighbor Number (k) with Fixed Spatial Neighbor Number (r = 3). a) Spatial plots of the simulated three‐omics dataset with k = 5, 10, 15, 20, 25, 30. b) Clustering results of the simulated three‐omics dataset. c) Line plots of clustering metrics for the simulated three‐omics dataset. d) Radar plots of clustering metrics for the simulated three‐omics dataset. e) Spatial plots of the human lymph node A1 dataset with k = 5, 10, 15, 20, 25, 30. f) Clustering results of the human lymph node A1 dataset. g) Line plots of clustering metrics for the human lymph node A1 dataset. h) Radar plots of clustering metrics for the human lymph node A1 dataset.

We fixed the spatial neighbor number at r = 3 and scanned the feature neighbor number k = 5, 10, 15, 20, 25, 30. In the simulated three‐omics dataset, clustering performance reached near perfection at k = 20 (**Figure** **8**c) with NMI ≈ 1.0, AMI ≈ 1.0, ARI ≈ 1.0, FMI ≈ 1.0, and comp ≈ 1.0. Moreover, overall performance remained stable across the range k = 5–30, indicating a high robustness of the dataset to feature graph construction. In the human lymph node A1 dataset, clustering performance peaked at k = 20–25 (Figure 8e), with NMI ≈ 0.409–0.410, AMI ≈ 0.406–0.407, and ARI ≈ 0.316–0.318, while FMI, V‐measure, and comp metrics showed similar trends. Smaller or larger k values led to decreased performance, suggesting that a moderate number of feature neighbors effectively captures cross‐modality similarity, whereas too few or too many neighbors may introduce information dilution or graph fragmentation.

Additionally, we fixed the feature neighbor number at k = 20 and scanned the spatial neighbor number r = 2–10. In the simulated three‐omics dataset, clustering performance reached near‐perfect levels at r = 3 (Extended Figure S6c, Supporting Information) with NMI ≈ 1.0, AMI ≈ 1.0, and ARI ≈ 1.0. Performance slightly decreased when r exceeded 6, but remained overall high, indicating strong robustness to spatial graph construction. In the human lymph node A1 dataset, clustering performance peaked at r = 3–5 (Extended Figure S7c, Supporting Information) with NMI ≈ 0.406–0.410, AMI ≈ 0.403–0.408, and ARI ≈ 0.316, and gradually declined beyond this range (e.g., r = 10, NMI ≈ 0.401, ARI ≈ 0.297), suggesting that overly large spatial neighborhoods introduce redundant signals from distant cells, thereby weakening the local tissue structure information.

From a graph neural network perspective, a small spatial neighbor number (r≈3) effectively captures local tissue context while avoiding oversmoothing, which could mix signals from biologically distinct regions. A moderate feature neighbor number (k≈20) balances cross‐cell feature aggregation and information dilution. The human lymph node A1 dataset exhibited higher sensitivity to k and r, likely due to intrinsic noise and modality heterogeneity, whereas the simulated three‐omics dataset demonstrated stronger stability, probably because of clearer tissue structure and higher modality concordance.

In summary, SpaBalance consistently achieved optimal or near‐optimal performance at r = 3 and k = 20 across both simulated and real tissue datasets. Even with deviations from these hyperparameter values, the method maintained stable performance, demonstrating robust applicability to diverse spatial multi‐omics datasets without requiring extensive parameter tuning.

## 3 Discussion

SpaBalance is an innovative spatial multi‐omics balancing learning framework that integrates different omics data effectively through a multi‐omics balancing learning mechanism and a dual learning strategy. It balances learning between omics while retaining omics‐specific information, thus improving the analytical precision and interpretative power of spatial multi‐omics data. Through its multi‐omics balancing learning mechanism, SpaBalance can adaptively learn and weigh the importance of different omics modalities, precisely capturing the interdependencies between spatial positions and omics features within each omics. This feature makes SpaBalance more expressive and biologically interpretable in spatial multi‐omics analysis, providing a more comprehensive and precise solution for multi‐omics integration analysis. The dual learning strategy aims to improve the learning precision of cross‐omics shared expressions while ensuring that omics‐specific information is retained. Specifically, the dual learning involves shared learning across omics and private learning within each omics. Shared learning minimizes the distance between omics, enhancing the collaborative expression across different omics and increasing the consistency of cross‐omics information, while private learning focuses on preserving each omics' unique information to ensure no loss of key omics‐specific features during the cross‐omics integration process. This strategy effectively prevents over‐integration of information across omics, maintaining the precision of multi‐omics data integration, and significantly improves the model's performance in multi‐omics analysis. Furthermore, the flexible architecture design of SpaBalance demonstrates excellent scalability and adaptability. Its multi‐level design breaks the integration process into two stages: within‐omics integration and between‐omics integration. Within‐omics integration uses a lightweight network design to enhance single‐omics feature learning and spatial information embedding, while also improving the model's robustness and spatial feature capture ability through data augmentation techniques. Between‐omics integration introduces a multi‐head attention mechanism to efficiently capture complex interactions between different omics and spatial positions, further enhancing the model's ability to model both local and global dependencies in multi‐omics data. This modular design not only supports the seamless expansion of multi‐omics data but is also compatible with various spatial omics technology platforms. Compared with existing methods, SpaBalance improves spatial multi‐omics data integration results and significantly enhances model scalability and application versatility through its multi‐omics balancing learning algorithm and modular design.

In the integration experiment with the human lymph node dataset, SpaBalance demonstrated superior performance compared to other tools and further validated the critical role of the multi‐omics balancing learning mechanism in optimizing balance between different omics, thus enhancing the integration of multi‐omics data. In the largest test dataset (spatial epigenome‐transcriptome mouse brain data with 9752 spatial points), models were run on a server equipped with an NVIDIA RTX A6000 GPU. The results showed that SpaBalance, compared to other methods, was able to resolve more refined cortical hierarchical structures. Additionally, in the quantitative analysis using RNA and ATAC omics‐specific labels, SpaBalance's dual learning strategy effectively preserved the key omics‐specific information of RNA and ATAC. Notably, in terms of computational efficiency, MOFA+, MultiVI, TotalVI, and PRAGA required hours to run, with MOFA+ and PRAGA taking the longest time, up to several hours, while STAGATE could not run due to high memory consumption. SpaBalance and SpatialGlue, however, completed the integration in just a few minutes, further validating the efficiency of SpaBalance. Furthermore, a systematic benchmark test using a simulated three‐omics dataset showed that SpaBalance significantly outperformed six advanced single‐omics and non‐spatial methods in multi‐omics data integration and spatial feature analysis, fully verifying its accuracy and effectiveness in spatial multi‐omics integration tasks. A direct comparison with current spatial multi‐omics methods (such as SpatialGlue and PRAGA) further demonstrated that SpaBalance can capture more intricate histological features and significantly enhance the analysis of complex spatial structures, thereby providing more accurate solutions for multi‐omics spatial omics research.

Although SpaBalance demonstrates excellent performance in multi‐omics integration, several limitations remain. First, SpaBalance requires preprocessing and dimensionality reduction of each omics to a common dimension; when one omics has significantly lower feature dimensionality than others, this uniform constraint may lead to information loss. Currently, protein omics often become a bottleneck due to technical limitations such as surface protein detection. Second, SpaBalance primarily focuses on spatial multi‐omics integration, whereas many biological processes, such as development or disease progression, exhibit substantial temporal dynamics. In the future, SpaBalance could be extended to spatiotemporal multi‐omics modeling by incorporating temporal graph neural networks or sequential Transformer modules, embedding spatial multi‐omics data from different time points into a unified spatiotemporal graph to capture dynamic biological processes. In addition, integrating large Transformers^[^ ^49^ ^]^ or multimodal pre‐trained models could enhance cross‐omics feature representation and long‐sequence modeling, enabling efficient modeling of complex global spatial–omics relationships and supporting in‐depth analysis of gene regulatory networks, protein interaction networks, and multi‐level biological information. This strategy would not only facilitate spatiotemporal dynamic analysis but also fully leverage the potential of large models in multi‐omics integration, providing a practical and scalable solution for future studies of complex biological systems.

## 4 Experimental Section

### The Overall Architecture of SpaBalance

SpaBalance is a novel spatial multi‐omics integration framework based on graph neural networks. Aiming to resolve the challenges posed by heterogeneous molecular omics and complex tissue architectures, SpaBalance employs a multi‐level feature interaction mechanism and a dynamic balance learning strategy to harmonize spatial topology with molecular specificity.

To accommodate dual‐omics datasets, the input features are defined as $X_{1}\in R^{N\times d_{1}}$ and $X_{2}\in R^{N\times d_{2}}$, where 𝑁 denotes the number of spatial locations and *d*_1_,*d*_2 _ represent the feature dimensions of each omics, such as transcriptomic and epigenomic or proteomic data. The framework maps these heterogeneous features into a unified latent space that preserves spatial co‐localization and functional complementarity. The overall architecture of SpaBalance consists of the following five core modules: 1) multi‐omics data augmentation module, 2) intra‐omics spatial and feature integration module, 3) cross‐omics multi‐head attention integration module, 4) dual learning module for intra‐ and cross‐omics representation, 5) multi‐omics balanced learning Module. These modules work in synergy to enable SpaBalance to achieve high‐fidelity integration of spatial multi‐omics data, supporting downstream tasks such as spatial domain identification, functional region annotation, and multi‐modal biomarker discovery. The detailed information of each module is described next.

### Neighborhood Graph Construction

In complex tissue samples, the spatial distribution of cells is not always uniform. Typically, spatially adjacent points tend to have similar cell types or cell states, so spatial information can be used to capture local cell relationships. However, relying solely on spatial position might overlook certain important biological features, as points with the same cell type or cell state may be far apart in space and cannot be directly captured through spatial neighborhood relationships. In addition to constructing the spatial graph *G_s_* =  (*V_s_*,*E_s_*) to reflect the spatial adjacency of cells, the similarity of cell features in the latent space also needs to be simulated using the feature graph *G_f_* =  (*V_f_*,*E_f_*), thereby compensating for the limitations of spatial information and comprehensively capturing cell relationships.

### Spatial Graph *G_s_* = (*V_s_*,*E_s_*)

The spatial graph represents the spatial adjacency relationship of cells in the tissue. Each point was considered a cell in space, where the node set *V_s_* represents all cells, and the edge set *E_s_* represents the adjacency relationships between cells. The adjacency relationship was defined by calculating the Euclidean distance between points. Specifically, for a given cell $i\in V_{s}$, the *r* nearest cells in space were selected as its neighbors. If the distance between two cells falls within the range of the nearest *r*  =  3 neighbors, the corresponding position in the adjacency matrix $A_{s}\in R^{N\times N}$ is set to 1, otherwise it is set to 0.

### Feature Graph *G_f_* = (*V_f_*,*E_f_*)

The feature graph captures the similarity of cells in the feature space. The node set *V_f_* represents all points in the cell feature space, and the edge set *E_f_* represents the similarity relationships between cells in the feature space. First, principal component analysis (PCA) was performed on the data of each omics to extract key features. Then, the k‐nearest neighbors (KNN) algorithm was applied in the PCA embedding space. For each cell $i\in V_{f}$, the *k*  =  20 nearest cells in the feature space were selected as its neighbors. If cell *j* is one of the k‐nearest neighbors of cell *i* in the feature space, the corresponding position in the adjacency matrix of the feature graph $A_{f}\in R^{N\times N}$ is set to 1, otherwise it is set to 0. By combining the spatial graph *G_s_* and the feature graph *G_f_*, the multidimensional cell relationships between space and features were captured more comprehensively.

### Data Augmentation

Data augmentation plays a crucial role in self‐supervised contrastive learning by improving model robustness, preventing overfitting to specific spatial graph structures or gene expression features, and helping the model learn a more general and stable representation of cell relationships. After constructing the adjacency graph, positive and negative samples were generated through data augmentation to assist the contrastive learning task. Specifically, given an adjacency graph *G*  =  (*V*, *E*) and a gene expression matrix $X\in R^{N\times F}$ (where *N* is the number of spatial points and *F* is the dimensionality of gene features), a random permutation operation was performed on the gene expression matrix *X* while keeping the topology of the adjacency graph unchanged. This results in a perturbed gene expression matrix $X^{\prime }=\left\{x_{1}^{\prime },x_{2}^{\prime },.,x_{N}^{\prime }\right\}\in R^{N\times F}$, constructing a new perturbed graph *G*′ =  (*V*′, *E*′). Here, the node and edge sets remain the same as the original graph, i.e., *V*′ =  *V* and $\;E^{\prime }=E$. Therefore, the regularized adjacency matrix $\tilde{A}\in R^{N\times N}$ remains identical to the original graph, with only the gene expression matrix being replaced by the randomly permuted *X*′.

This data augmentation method disrupts the feature information of spatial points while generating perturbed data that was similar to the original graph. This helps the model distinguish true biological patterns from random noise in contrastive learning, thereby improving generalization ability and understanding of complex spatial relationships.

### Intra‐Omics Integration‐Graph Neural Network‐Based Encoding for Spatial and Feature Representations

A Graph Neural Network (GNN) encoder was used to learn representations of cellular spots, capturing important aspects of gene expression profiles and spatial location information. The encoder takes an adjacency graph *G* and a normalized gene expression matrix *X* as input and learns latent representations *Z_i_* for each cell point *i* through a Graph Convolutional Network (GCN). This was achieved by iteratively aggregating representations from neighboring nodes to update the latent representation at each layer.

The representation at the *l*‐th layer of the encoder is given by:

(1) $$\;Z^{l}=\sigma \left(\hat{A}Z^{l-1}W^{l-1}+b^{l-1}\right)$$

where $\hat{A}=D^{-\frac{1}{2}}\;AD^{\frac{1}{2}}$ is the normalized adjacency matrix, *D* is the degree matrix, *A* is the adjacency matrix of the graph, *W* ^*l* − 1^ and *b* ^*l* − 1^ are trainable weight matrices and bias vectors, respectively, σ(·) is a non‐linear activation function (e.g., ReLU), *Z* ^0^ is the input gene expression matrix, Each layer's output *Z^l^* represents the latent representation of nodes.

To capture both spatial and feature‐based information, separate encoding processes was designed for the spatial adjacency graph *G_s_* and the feature similarity graph *G_f_*. The spatial graph reflects the adjacency of cells in space, while the feature graph captures similarity relationships between cells in phenotypic space. This enables the encoder to aggregate information from neighboring nodes via graph convolutions, capturing different local patterns and dependencies.

For each omics, the encoder performs GCN operations separately on *G_s_* and *G_f_*. The representations at the *l*‐th layer are computed as follows:

Spatial Graph Representation:

(2) $$H_{s}^{l}=\sigma \left({\hat{A}}_{s}H_{s}^{l-1}W_{s}^{l-1}+b_{s}^{l-1}\right)$$

Feature Graph Representation:

(3) $$H_{f}^{l}=\sigma \left({\hat{A}}_{f}H_{f}^{l-1}W_{f}^{l-1}+b_{f}^{l-1}\right)$$

where ${\hat{A}}_{s}$ and ${\hat{A}}_{f}$ are the normalized adjacency matrices of the spatial and feature graphs, respectively, $W_{s}^{l-1}$ and $W_{f}^{l-1}$ are trainable weight matrices, $b_{s}^{l-1}$ and $b_{f}^{l-1}$ are bias vectors, $H_{s}^{l}$ and $H_{f}^{l}$ represent the learned latent representations from the spatial and feature graphs, respectively.

The final encoder outputs: $H_{s}^{L}\in R^{N\times d_{latent}}$, representing the local environmental features of cell spatial positions, capturing physical adjacency and microenvironment information, $H_{s}^{L}\in R^{N\times d_{latent}}$, representing feature‐based cell relationships that capture molecular similarity among non‐adjacent spatial points. These two latent representations complement each other, encoding different semantic and topological information. They jointly characterize the complex data features of a single omics(e.g., RNA, ADT, or ATAC). To create a comprehensive representation for each spot, the two latent representations along the feature dimension were concatenated:

(4) $$x=concat\left(H_{s}^{L},H_{f}^{L}\right)\in R^{N\times \left(2\times d_{latent}\right)}$$

### Feature Transformation and Integration

A Multi‐Layer Perceptron (MLP) with a Dropout mechanism was used to transform and fuse the concatenated representations. This MLP consists of multiple linear layers, non‐linear activation functions, Dropout layers, and an optional LayerNorm layer. The goal was to map high‐dimensional features into a more compact and expressive latent space, producing the final intra‐omics representation.

For an input representation 𝑥, each layer of the MLP is computed as:

(5) $$x^{\left(i\right)}=Dropout\left(\sigma \left(W_{i}x^{i-1}+b_{i}\right)\right)$$

where *x* ^(*i*)^ is the output of the i‐th layer, *W_i_* and *b_i_* are learnable parameters (weights and biases). Dropout was a regularization technique that randomly zeroes out neurons to prevent overfitting, *x* ^(0)^ =  *x* is the input to the MLP.

After passing through *L* layers of the MLP, the final intra‐omics fused representation was obtained:

(6) $$H_{final}=x^{\left(L\right)}\;\in R^{N\times d_{hidden}}$$

where *d_hidden_* is the dimensionality of the hidden features. This representation integrates multi‐level semantic information from both spatial and feature graphs, providing a comprehensive description of each spot's characteristics. It serves as the input for downstream tasks such as classification, regression, or generative modeling.

### Inter‐Omics Integration

Each omics can only capture partial characteristics of complex biological samples. Therefore, effective multi‐omics data integration was required to comprehensively and accurately describe the biological information of the samples. Since different omics may be both complementary and divergent, assigning different weights based on each omics' contribution to the overall representation was necessary to achieve a more reasonable integration. To this end, a multi‐head cross‐omics attention mechanism^[^ ^50^ ^]^ was adopted to adaptively fuse feature representations from different omics. The multi‐head mechanism allows the model to learn diverse inter‐omics associations from different subspaces, capturing complex cross‐omics dependencies and enhancing the model's understanding of multi‐omics information. Cross‐omics attention enables interaction modeling between omics and dynamically evaluates each omics' relative importance, thereby assigning adaptive weights based on the omics contribution distribution.

### Learning Omics‐Specific Weights

For the unified latent representations $H_{final\;}^{\left(1\right)}$ and $\;H_{final}^{\left(2\right)}$ from different omics, a linear transformation was first applied to extract features and generate Query (Q), Key (K), and Value (V) representations to model inter‐omics interactions:

(7) $$Q=W_{Q}\;H_{final}^{\left(1\right)},K=W_{K}\;H_{final}^{\left(2\right)},V=W_{V}\;H_{final}^{\left(2\right)}$$

where *W_Q_* 、*W*_*K*_ and *W_V_* are learnable linear mapping matrices, and the shapes of *Q*, *K*, *V* are: $Q,K,V\in R^{N\times d_{all_{head}}}$.

Next, *Q*, *K*, *V* was reshaped into the multi‐head attention format, where each head has a dimension of $d_{h_{size}}=\frac{d_{all_{head}}}{head_{num}}$, resulting in: $Q,K,V\in R^{head_{num}\times N\times d_{h_{size}}}$. For each cell 𝑖 and each omics 𝑚, mean aggregation was performed on the multi‐head attention outputs to obtain omics‐specific feature representations:

(8) $$H_{attn}^{\left(n\right)}=Attention\;\left(Q_{n},K_{n},V_{n}\right)=Softmax\left(\frac{Q_{n}\cdot K_{n}^{T}}{\sqrt{d_{h\_size}}}\right)V_{n}$$

(9) $$H_{mean}=\frac{1}{head\_num}\;\sum_{n=1}^{head\_num}H_{attn}^{\left(n\right)}$$

Then, a omics‐specific linear transformation layer was used to generate an attention score $g_{m}^{i}$ for each omics:

(10) $$g_{m}^{i}=v_{m}^{T}\;\cdot \tanh\left(W_{m}H_{mean}^{i}+b_{m}\right)$$

where *v_m_*, *W_m_* and *b_m_* are learnable parameters associated with omics 𝑚, and $g_{m}^{i}$ represents the importance of omics 𝑚 for sample 𝑖.

To ensure the attention scores across different omics have a probabilistic meaning, a softmax function was applied to normalize them, yielding the weight $\beta_{m}^{i}$ for each omics:

(11) $$\beta_{m}^{i}=\frac{\exp\left(g_{m}^{i}\right)}{\sum_{m=1}^{M}\exp\left(g_{m}^{i}\right)}$$

where 𝑀 is the total number of omics, and $\beta_{m}^{i}$ represents the normalized attention coefficient of omics 𝑚 for sample 𝑖, reflecting its contribution to the final representation.

### Generation of Multi‐Omics Latent Representations

After computing the weight for each omics, a weighted sum of each omics' latent representation was performed to generate the final multi‐omics latent representation 𝑍:

(12) $$z_{i}=\sum_{m=1}^{M}\beta_{m}^{i}\cdot H_{final}^{\left(m\right)}$$

where $z_{i}\in R^{d\_hidden}$ is the multi‐omics integration representation for sample 𝑖. The final multi‐omics representation matrix 𝑍$\in R^{N\times d_{hidden}}$ can be used for various downstream analysis tasks such as cell clustering, data visualization, and differential gene expression analysis.

### Omics Learning

The model adopts a dual learning strategy to ensure the precise alignment of cross‐omics shared information while preserving the omics‐specific features of each dataset. This dual learning approach consists of intra‐omics private learning and inter‐omics shared learning.

### Intra‐Omics Private Learning

Intra‐omics private learning ensures that the integrated multi‐omics representation 𝑍 shares expression patterns across omics while retaining omics‐specific information. To achieve this, 𝑍 was guided to maintain a mapping relationship with the original normalized expression space and incorporate self‐supervised contrastive learning^[^ ^25^ ^]^ (SCL) to enhance the model's ability to distinguish omics‐specific features.

To further reinforce this mapping relationship, independent decoders was designed for each omics, enabling the integrated multi‐omics representation 𝑍 to effectively reconstruct the original expression profiles. Specifically, the model learns a mapping from 𝑍 back to the original feature space, ensuring that while fusing multi‐omics information, it still accurately captures the expression characteristics of each omics. To achieve this objective, a learning process was introduced that minimizes the discrepancy between the reconstructed features and the original features, constraining the optimization of 𝑍 to preserve both global information integration and local omics specificity. The reconstructed expression at the 𝑙‐th layer (l∈{1,2,…,L−1,L}) is computed as follows:

(13) $${\hat{H}}^{l}=\sigma \left({\hat{A}}_{s}Z^{l-1}W_{d}^{l-1}+b_{d}^{l-1}\right)$$

where $W_{d}^{l-1}$ and $b_{d}^{l-1}$ are trainable weight matrices and bias vectors, and 𝜎 is the activation function. To optimize this learning process, a reconstruction loss function that minimizes the difference between the reconstructed features and the original features was defined:

(14) $$L_{recon}=\sum_{i=1}^{N}{\left\|x_{i}-{\hat{h}}_{i}\right\|}_{F}^{2}$$

where *x*_i_ represents the original features of sample 𝑖, ${\hat{h}}_{i}$ denotes the corresponding reconstructed features, and 𝑁 is the number of samples.

This optimization strategy ensures that while learning omics‐shared information, 𝑍 still preserves the unique expression patterns of each omics, providing a more precise feature representation for downstream spatial multi‐omics integration. To enhance the information richness and discriminative power of the spatial representation *H_s_*, self‐supervised contrastive learning was employed to ensure the model captures the local contextual information of spatial positions. Specifically, a graph neural network (GNN) encoder was used to generate spatial position representations from both the original graph 𝐺 and the perturbed graph *G*′, producing spatial position representation matrices *H_s_* ∈*R* ^*N* × *d*^ and $H_{s}^{\prime }\in R^{N\times d}$, respectively. The original and perturbed graphs were derived from the same omics, and different data views were constructed for representation learning. Since this constraint was applied within each omics, it ensures that spatial relationships within a single omics remain more compact and consistent. In SCL, for each spatial position 𝑖, the local environment vector *g_i_* was computed by aggregating the mean representation of its neighboring points, capturing the microenvironment information of spatial positions. During contrastive learning:

Positive samples: Representation $h_{s}^{i}$ of position 𝑖 in the original graph, paired with its local environment vector *g_i_*.

Negative samples: Representation 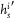 of position 𝑖 in the perturbed graph, paired with the local environment vector *g_i_* from the original graph.

Is defined.

The model was trained to maximize the mutual information of positive sample pairs while minimizing the mutual information of negative sample pairs, enhancing the similarity of adjacent spatial positions while increasing the distinction between non‐adjacent positions. This design effectively captures the local structure of spatial data, ensuring higher spatial representation consistency and better discriminative power within a single omics. The SCL loss function is defined as:

(15)

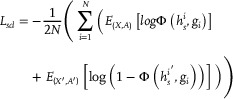

where *X* and *A* denote the normalized gene expression profile and the normalized adjacency matrix corresponding to *H_s_*, respectively. Similarly, 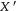 and 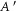 correspond to 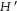. Φ(·) was a discriminator that distinguishes between positive and negative sample pairs, outputting the probability of a pair being a positive match. To improve model stability, a symmetric contrastive loss for the perturbed graph *G*′is defined:

(16)

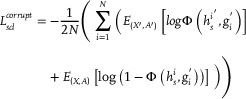




This symmetric loss ensures that the model learns local spatial information not only from the original graph but also from the perturbed graph, enhancing the robustness of spatial representations. This contrastive learning strategy enforces higher similarity for spatially adjacent locations, while maintaining distinct representations for non‐adjacent locations, effectively capturing the local structure and biological characteristics of spatial data. The final intra‐omics loss combines reconstruction loss and omics‐specific contrastive learning loss, defined as:

(17) $$L_{omics}=\lambda_{1}\;L_{recon}+\lambda_{2}\left(L_{scl}+L_{scl}^{corrupt}\right)$$

where λ_1_ and λ_2_ are hyperparameters that balance the contributions of different loss components, ensuring that the model maintains a trade‐off between feature reconstruction and representation distinctiveness.

### Inter‐Omics Shared Learning

To ensure alignment between different omics in the shared latent space, a cross‐omics learning mechanism that includes consistency alignment learning and Barlow Twins contrastive learning was designed to enhance the quality of feature representations in the shared space. Although the model can learn omics‐specific representations through intra‐omics optimization, learning strategies applied separately to each omics do not guarantee that the representation manifolds of different omics remain consistent in the shared space. Due to differences in measurement techniques and biological properties, direct integration may lead to misalignment between omics‐specific feature distributions, thereby affecting the effectiveness of data integration. To address this issue, a cross‐omics consistency learning strategy was employed. The core objective was to constrain the mapping relationship between the representation of one omics and the corresponding representation generated through the decoder‐encoder pathway of another omics, ensuring that data from different omics remain aligned in the shared space. Specifically, let Y be the latent representation extracted from a given omics, and let *Y*′ be the corresponding representation generated through the decoder‐encoder pathway of another omics. By optimizing this mapping relationship, the original representation and the cross‐omics generated representation are encouraged to become more consistent, thereby enhancing semantic alignment across omics. This process was formulated as follows:

(18) $${\hat{Y}}_{1}^{l}=\sigma \left({\hat{A}}_{s}\left(\sigma \left({\hat{A}}_{s}Y_{1}^{l-1}W_{d2}^{l-1}+b_{d2}^{l-1}\right)\right)W_{e2}^{l-1}+b_{e2}^{l-1}\right)$$

(19) $${\hat{Y}}_{2}^{l}=\sigma \left({\hat{A}}_{s}\left(\sigma \left({\hat{A}}_{s}Y_{2}^{l-1}W_{d1}^{l-1}+b_{d1}^{l-1}\right)\right)W_{e1}^{l-1}+b_{e1}^{l-1}\right)$$

(20) $$L_{corr}=\gamma_{1}\;\sum_{i=1}^{N}{\left\|y_{i}^{1}-{\overset{⌢}{y}}_{i}^{1}\right\|}_{F}^{2}+\gamma_{2}\sum_{i=1}^{N}{\left\|y_{i}^{2}-{\overset{⌢}{y}}_{i}^{2}\right\|}_{F}^{2}$$

where $y_{i}^{1}$ and $y_{i}^{2}$ denote the representations of omics 1 and omics 2, respectively, while ${\hat{y}}_{i}^{1}$ and ${\hat{y}}_{i}^{2}$ are the corresponding cross‐omics representations generated through the pathways of omics 2 and omics 1, respectively. *W_d_* and *W_e_* are trainable parameters of the decoder and encoder, respectively. Optimizing this process ensures that the mapping relationships between different omics in the shared space remain stable, improving the reliability of data integration.

To further enhance alignment in the shared omics space, the Barlow Twins contrastive learning method was adopted. This approach encourages consistency between the latent representations of different omics while reducing redundancy, ensuring effective cross‐omics integration. For the latent representations ${\hat{H}}_{1}^{l}$ and ${\hat{H}}_{2}^{l}$ from two different omics, the contrastive loss is defined as:

(21) $$L_{bt}=\sum_{i=1}^{d}{\left(C_{ii}-1\right)}^{2}+\gamma_{3}\sum_{i=1}^{d}\sum_{j\neq i}{C_{ij}}^{2}$$

where $C=\frac{{\left({\hat{H}}_{1}^{l}\right)}^{T}{\hat{H}}_{2}^{l}}{N}$ represents the cross‐correlation matrix between the latent representations of the two omics modalities, with *N* being the number of samples and *d* being the feature dimension. *C_ii_* (diagonal elements) are constrained to approach 1 to ensure alignment of corresponding features. *C_ij_* (off‐diagonal elements) are constrained to approach 0 to reduce redundancy between modalities. γ_3_ was a hyperparameter that balances the diagonal and off‐diagonal terms.

To further improve alignment, a bidirectional strategy was employed, swapping ${\hat{H}}_{1}^{l}$ and ${\hat{H}}_{2}^{l}$ to compute the mapping relationship in both directions and taking the average. The final optimization objective is:

(22) $$L_{multi\_omics}=L_{corr}\;+\frac{1}{2}\beta \left(L_{bt}\left({\hat{H}}_{1}^{l},\;{\hat{H}}_{2}^{l}\right)+L_{bt}\left({\hat{H}}_{2}^{l},\;{\hat{H}}_{1}^{l}\right)\right)$$

where β is a weight parameter that controls the strength of omics alignment. In the early training stages, as inter‐omics relationships are relatively simple, a fixed weight can be used. As training progresses, omics relationships become more complex, and β can be dynamically adjusted to fine‐tune the alignment. This design ensures that the model initially focuses on learning omics‐specific features, while gradually enhancing cross‐omics integration in later stages, ultimately improving the accuracy of multi‐omics data integration.

### Multi‐Omics Balanced Learning

In multi‐omics learning, different omics (such as gene expression and phenotypic features) have their own optimization objectives, which may inherently differ and lead to gradient conflicts. Traditional methods typically apply a fixed weighted summation of intra‐omics losses and inter‐omics losses. However, such a fixed weighting approach may cause the gradient of certain omics to dominate during training, suppressing the effective learning of other omics.^[^ ^51^ ^]^ Furthermore, the gradient directions of intra‐omics and inter‐omics objectives may contradict each other, leading to parameter updates that deviate from the optimal solution. This negatively impacts the model's ability to learn both omics‐specific features and cross‐omics joint representations.

To address these issues, a dynamic balancing strategy was proposed for multi‐omics learning. This approach adjusts the contributions of different losses in real‐time, ensuring that the model effectively learns omics‐specific features while also enhancing alignment in the shared cross‐omics space.

### Computing Intra‐Omics and Inter‐Omics Gradients

First, the gradient of each omics‐specific loss, denoted as $g_{i}^{s}$ (where $g_{1}^{s}$ and $g_{2}^{s}$ represent the gradients of omics 1 and omics 2, respectively), was computed. Additionally, the gradient of the joint inter‐omics loss, denoted as $g_{i}^{m}$, is computed. To evaluate the consistency between intra‐omics and inter‐omics gradient directions, cosine similarity, defined as was used:

(23) $$cos\beta =\frac{g_{i}^{s}\cdot g_{i}^{m}}{\left\|g_{i}^{s}\right\|\left\|g_{i}^{m}\right\|}$$

If cosβ≥0 (gradient directions are consistent), equal weights was assigned to intra‐omics and inter‐omics losses:

(24) $$\alpha_{i}^{m}=\alpha_{i}^{s}=0.5$$

If cos β < 0 (gradient directions conflict), the norm of the combined gradient was minimized to adjust the weights dynamically. The optimal weighting coefficients for intra‐omics and inter‐omics losses are computed as:

(25) $$\alpha_{i}^{m}=\frac{{\left(g_{i}^{s}-g_{i}^{m}\right)}^{T}g_{i}^{s}}{{\left\|g_{i}^{m}-g_{i}^{s}\right\|}^{2}},\alpha_{i}^{s}=1-\alpha_{i}^{m}$$

### Computing the Total Loss

Using the dynamically computed weights, the final total loss is determined by both intra‐omics and inter‐omics losses:

(26) $$L_{total}=\left(\frac{1}{n}\sum_{i=1}^{n}\alpha_{i}^{m}\right)\;L_{multi\_omics}+\sum_{i=1}^{n}\alpha_{i}^{s}L_{omics}$$

where $L_{multi\_omics}$ represents the inter‐omics joint loss (e.g., contrastive loss, correlation loss). *L_omics_* represents the individual omics‐specific loss (e.g., reconstruction loss, classification loss). $\alpha_{i}^{m}$ and $\alpha_{i}^{s}$ are the dynamically adjusted weights for the intra‐omics and inter‐omics losses for sample 𝑖.

By dynamically adjusting the weighting between intra‐omics and inter‐omics losses based on gradient similarity, this approach effectively coordinates the learning process across omics. It mitigates gradient conflicts, ensuring more stable optimization of shared parameters. This leads to enhanced feature integration in the multi‐omics space and improves overall model performance by achieving a better balance between omics‐specific learning and cross‐omics alignment.

### Implementation Details

In the training of all datasets, a learning rate of 0.0001, β = 0.5, λ_2_ = γ_1_ = γ_2_ = γ_4_ = 1, and γ_3_ = 0.005 was used. To accommodate differences in feature distributions across datasets, a specific weight factor λ_1_ for each dataset was empirically assigned: 120 for the SPOTS mouse spleen dataset, 30 for the 10x Genomics Visium human lymph node dataset, 60 for the Stereo‐CITE‐seq mouse thymus dataset, and 50 for the spatial epigenome‐transcriptome mouse brain dataset. These weight factors were used to adjust the contribution of each loss term, ensuring that the model could effectively capture multi‐omics features across different datasets. A detailed summary of all hyperparameters is provided in Table S1 (Supporting Information).

### Baseline Methods and Parameter Settings

All baseline methods were implemented following the official tutorials or recommended practices. For Seurat WNN, 2000 and 3000 highly variable genes (HVGs) were selected for RNA–protein and RNA–ATAC (histone) data, respectively, with log‐normalization applied. Dimensionality reduction was set to 30 (RNA) and 18 (protein) for RNA–protein integration, and 10 (RNA and ATAC/histone) for RNA–ATAC (histone) integration.

For totalVI and MultiVI, the scVI package (version 1.2.2) was used with preprocessing following the standard SCANPY workflow. Specifically, totalVI was trained on the top 4000 HVGs, while for MultiVI on RNA–epigenome (ATAC/histone) data, genes and peaks expressed in less than 1% of spots were removed. It was noted that totalVI was originally designed for CITE‐seq.

For MOFA+, the official implementation was used with a default latent dimensionality of 15, maximum of 500 iterations, and Gaussian likelihood for continuous features (RNA) and Poisson likelihood for count features (ATAC/protein). RNA and protein/ATAC/histone matrices were concatenated and provided as different modalities to learn a shared latent representation.

For SpatialGlue, the default training settings reported in the original paper (training epochs, batch size, learning rate) was adopteded. For PRAGA, the default settings provided in the released code was used. STAGATE, originally designed for unimodal spatial transcriptomics, was applied following the authors’ tutorial.

After model training, the latent representations from all methods (Seurat WNN, totalVI, MultiVI, MOFA+, SpatialGlue, PRAGA, STAGATE) were clustered using the mclust algorithm.

A detailed summary of baseline parameter settings is provided in Table S2 (Supporting Information).

## Conflict of Interest

The authors declare no conflict of interest.

## Author Contributions

Y.C., Y.Z. (Yuansong Zeng), Z.W. and H.Zhao. conceived and supervised the project. Y.Z.(Yong Zhao), and Y.C., contributed to the algorithm implementation. Y.Z. (Yuansong Zeng), Y.Z. (Yong Zhao), and Y.C. wrote the manuscript. Y.C., Y.Z. (Yong Zhao), C.Y., T.T., X.L., H.Zhang, H.Zhao, Z.W., and Y.Z. (Yuansong Zeng) were involved in the discussion and proofreading.

## Supporting information



Supporting Information

## Data Availability

The evaluated datasets are accessible through the papers cited, and detailed information about these datasets can be obtained by accessing https://github.com/nudt‐bioinfo/SpaBalance. The datasets used in this study are publicly available and come with curated ground truth annotations provided by the original dataset sources. Specifically, the ground truth labels for the simulated three‐omics dataset and human lymph node sample were obtained directly from the references cited in the respective datasets.
