# Supplementary material for: SpaBalance: Balanced Learning for Efficient Spatial Multi‐Omics Decoding
Source: Adv Sci (Weinh). 2025 Oct 14;12(48):e12973. doi: 10.1002/advs.202512973 (PMC12752582; doi:10.1002/advs.202512973)
Supplement: Supplementary file 1 — Supporting Information [file ADVS-12-e12973-s001.docx]

# Appendix


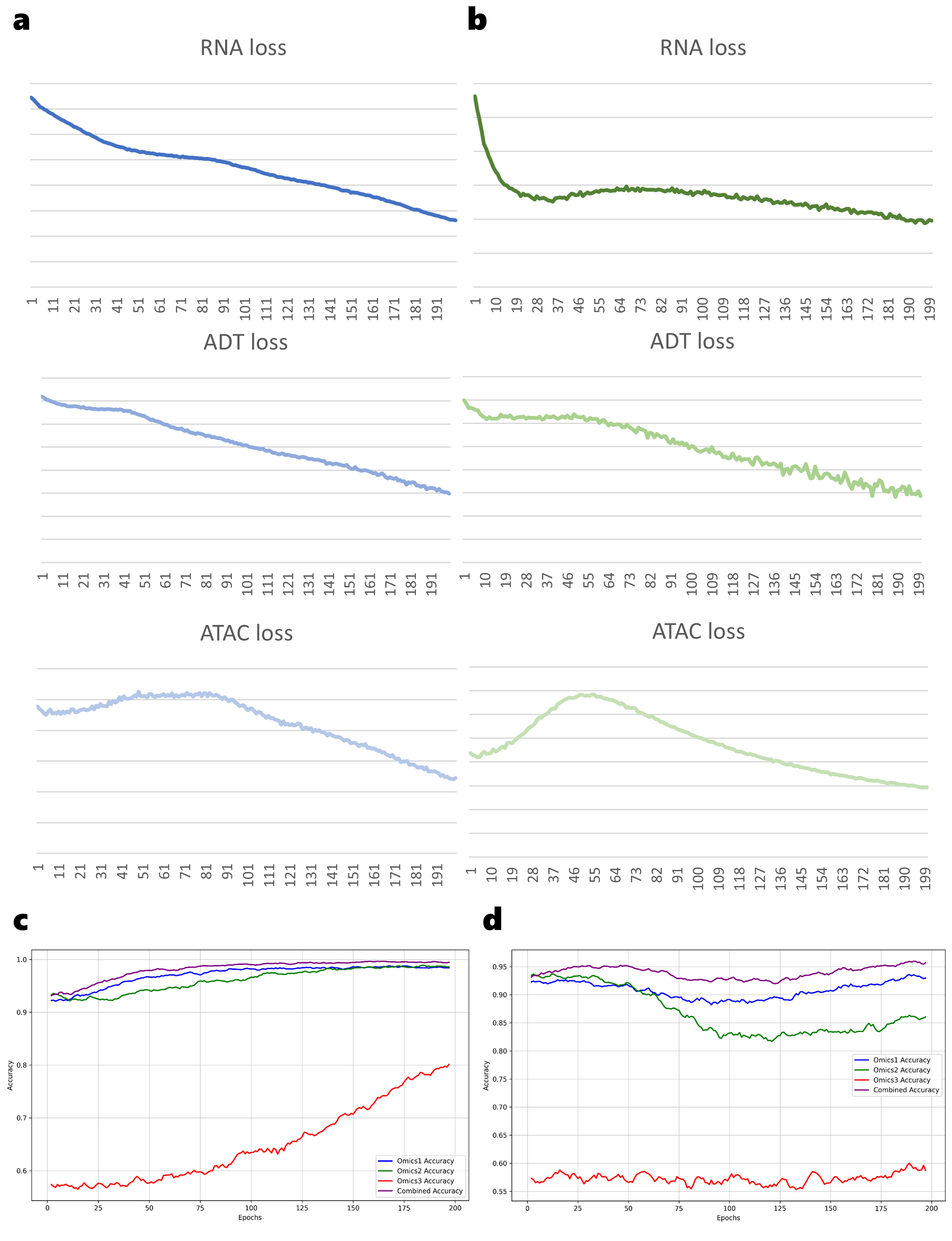


**Extended Fig. 1 | Supplementary Results of the Ablation Study on Simulated Three-omics Dataset Using SpaBalance.** a, Loss trend plots for RNA, ADT, and ATAC omics during SpaBalance training. b, Loss trend plots for RNA, ADT, and ATAC omics during SpaBalance training without multi-omics balanced learning. c, Accuracy trend plots for RNA, ADT, ATAC, and the integrated representation during SpaBalance training. d, Accuracy trend plots for RNA, ADT, ATAC, and the integrated representation during SpaBalance training without multi-omics balanced learning.


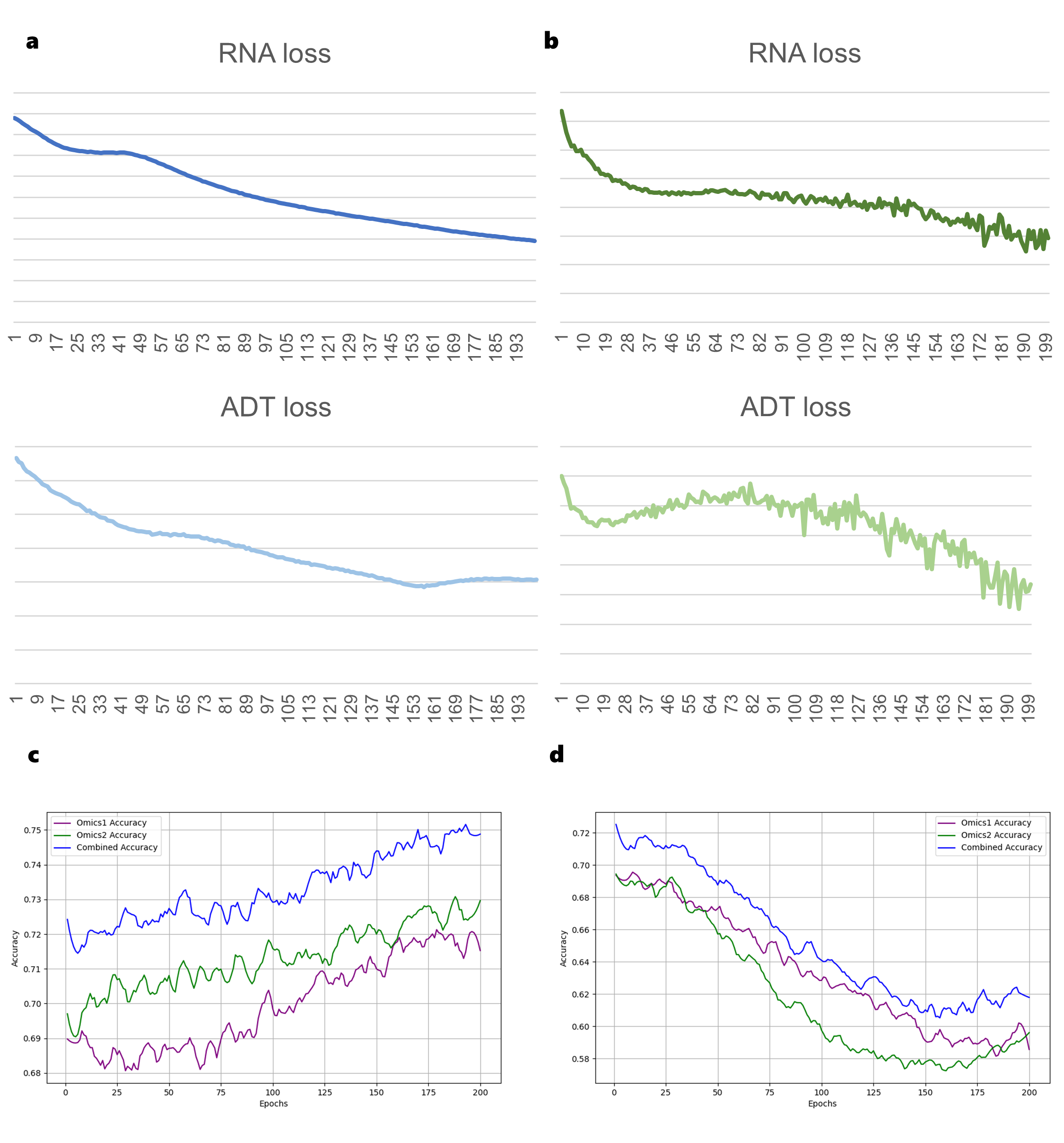


**Extended Fig. 2 | Supplementary Results of the Ablation Study on Human Lymph Node A1 Using SpaBalance.** a, Loss trend plots for RNA and ADT omics during SpaBalance training. b, Loss trend plots for RNA and ADT omics during SpaBalance training without multi-omics balanced learning. c, Accuracy trend plots for RNA, ADT, and the integrated representation during SpaBalance training. d, Accuracy trend plots for RNA, ADT, and the integrated representation during SpaBalance training without multi-omics balanced learning.


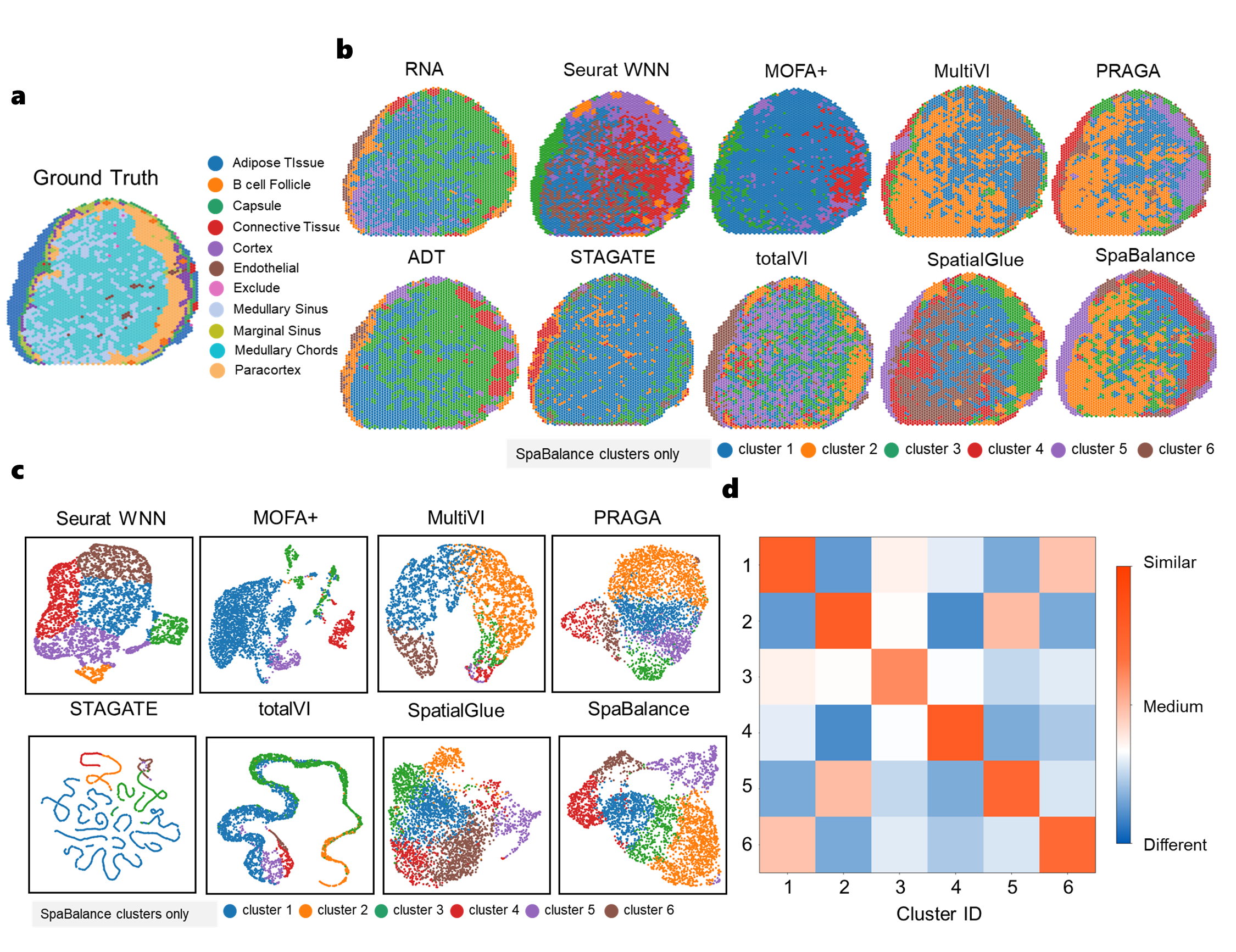


**Extended Fig. 3 | Integration Results on Human Lymph Node D1.** a, Manually annotated human lymph node sample D1 [28]. b, Spatial clustering visualizations of RNA and protein omics for sample D1, and clustering results on sample D1 using spatial multi-omics integration methods—STAGATE, Seurat WNN, totalVI, MultiVI, MOFA+, SpatialGlue, PRAGA, and SpaBalance. Note: Cluster colors do not directly correspond to the same captured structures across methods. c, Final representation clustering visualization results of STAGATE, Seurat WNN, totalVI, MultiVI, MOFA+, SpatialGlue, PRAGA, and SpaBalance. d, Heatmap of intra-cluster compactness and inter-cluster separability for SpaBalance on lymph node sample D1, computed using unsupervised Jaccard similarity.


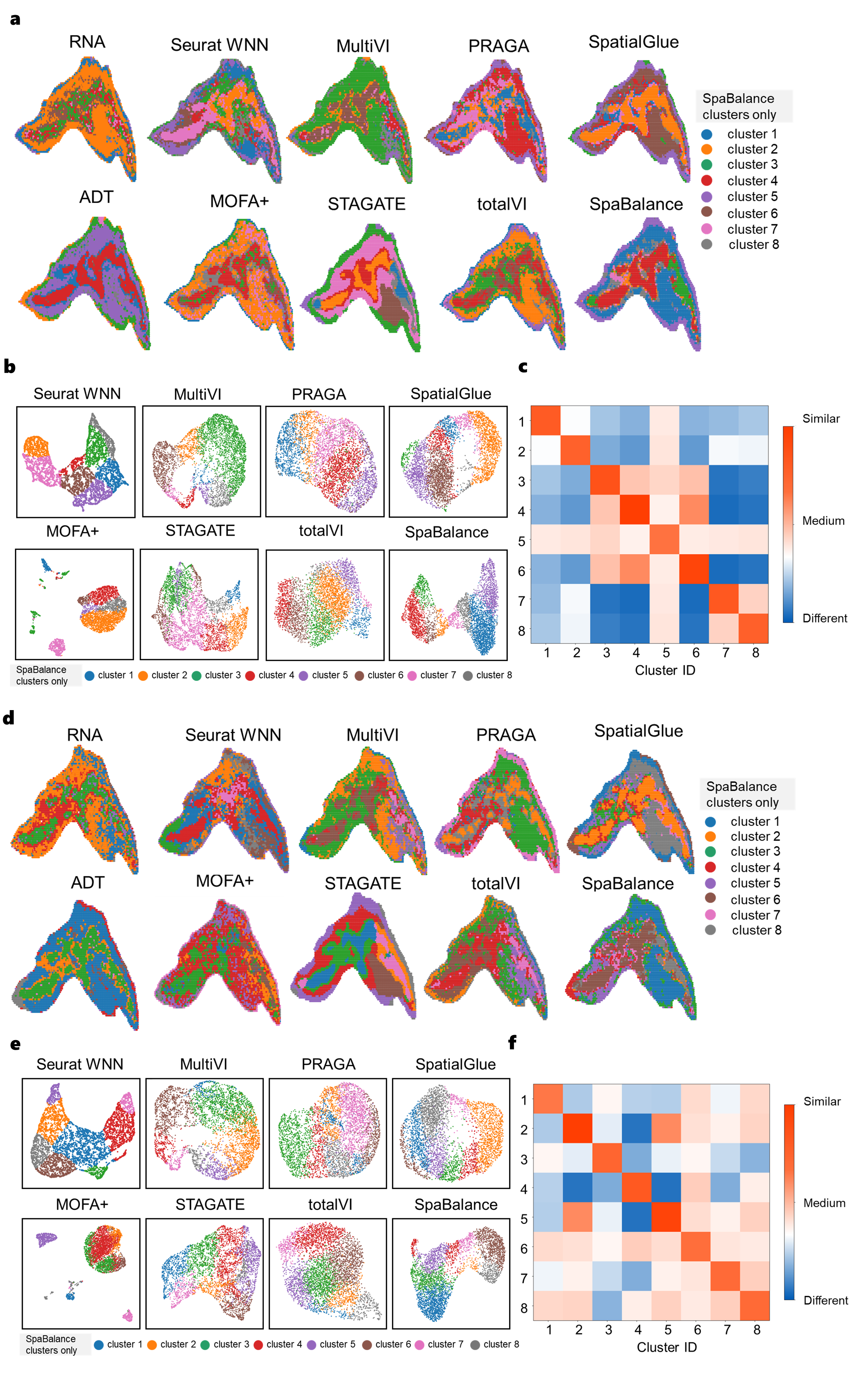


**Extended Fig. 4 | Integration of Mouse Thymus1 and Mouse Thymus2 Datasets.** a, Spatial clustering visualizations of RNA and protein omics for mouse thymus1, along with clustering results produced by spatial multi-omics integration methods—STAGATE, Seurat WNN, totalVI, MultiVI, MOFA+, SpatialGlue, PRAGA, and SpaBalance. Note: Cluster colors do not correspond to identical tissue structures across methods. b, Final representation clustering visualizations on mouse thymus1 for STAGATE, Seurat WNN, totalVI, MultiVI, MOFA+, SpatialGlue, PRAGA, and SpaBalance. c, Heatmap of intra-cluster compactness and inter-cluster separability for SpaBalance on mouse thymus1, calculated using unsupervised Jaccard similarity. d, Spatial clustering visualizations of RNA and protein omics for mouse thymus2, and clustering results using the same integration methods as above. e, Final representation clustering visualizations on mouse thymus2 for all integration methods. f, Heatmap of intra-cluster compactness and inter-cluster separability for SpaBalance on mouse thymus2, computed using unsupervised Jaccard similarity.


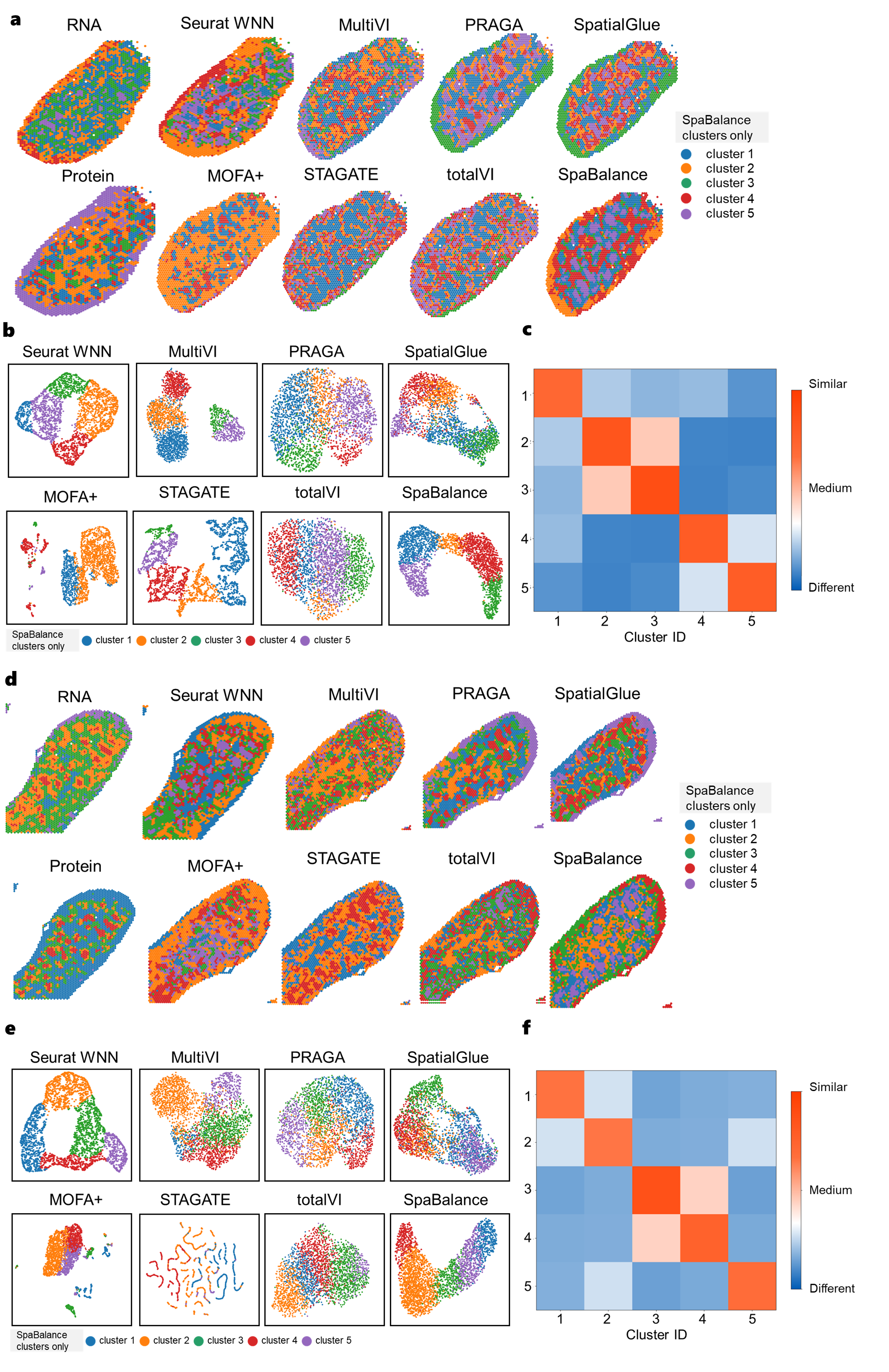


**Extended Fig. 5 | Integration of Mouse Spleen1 and Mouse Spleen2 Datasets.** a, Spatial clustering visualizations of RNA and protein omics for mouse spleen1, along with clustering results produced by spatial multi-omics integration methods—STAGATE, Seurat WNN, totalVI, MultiVI, MOFA+, SpatialGlue, PRAGA, and SpaBalance. Note: Cluster colors do not correspond to identical tissue structures across methods. b, Final representation clustering visualizations on mouse spleen1 for STAGATE, Seurat WNN, totalVI, MultiVI, MOFA+, SpatialGlue, PRAGA, and SpaBalance. c, Heatmap of intra-cluster compactness and inter-cluster separability for SpaBalance on mouse spleen1, calculated using unsupervised Jaccard similarity. d, Spatial clustering visualizations of RNA and protein omics for mouse spleen2, and clustering results using the same integration methods as above. e, Final representation clustering visualizations on mouse spleen2 for all integration methods. f, Heatmap of intra-cluster compactness and inter-cluster separability for SpaBalance on mouse spleen2, computed using unsupervised Jaccard similarity.


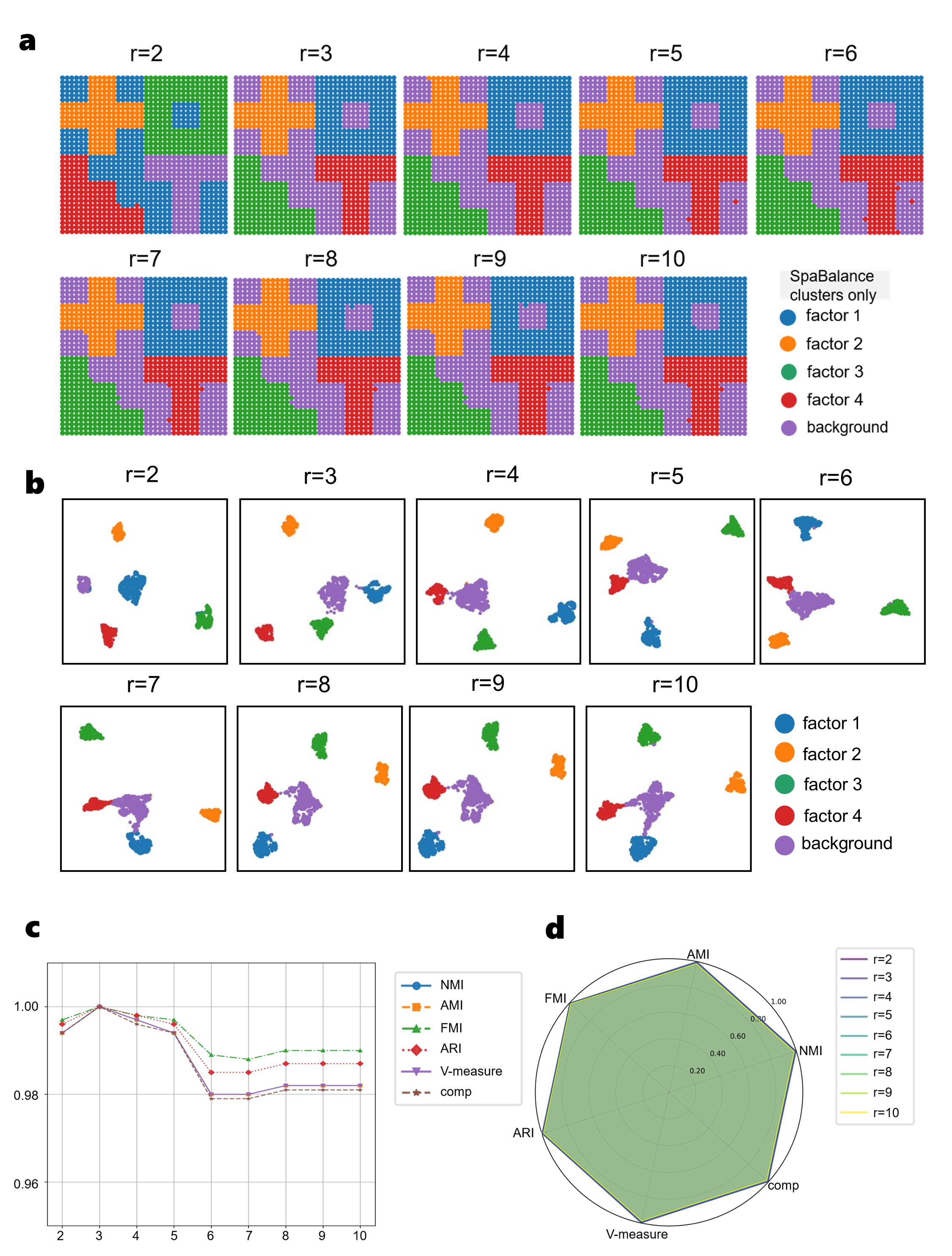


**Extended Fig. 6 | Performance of SpaBalance Under Varying Spatial Neighbor Number (r) with Fixed Feature Neighbor Number (k=20) in the Simulated Three-Omics Dataset.** a, Spatial plots with r=2, 3, 4, 5, 6, 7, 8, 9, 10. b, Clustering results. c, Line plots of clustering metrics. d, Radar plots of clustering metrics.


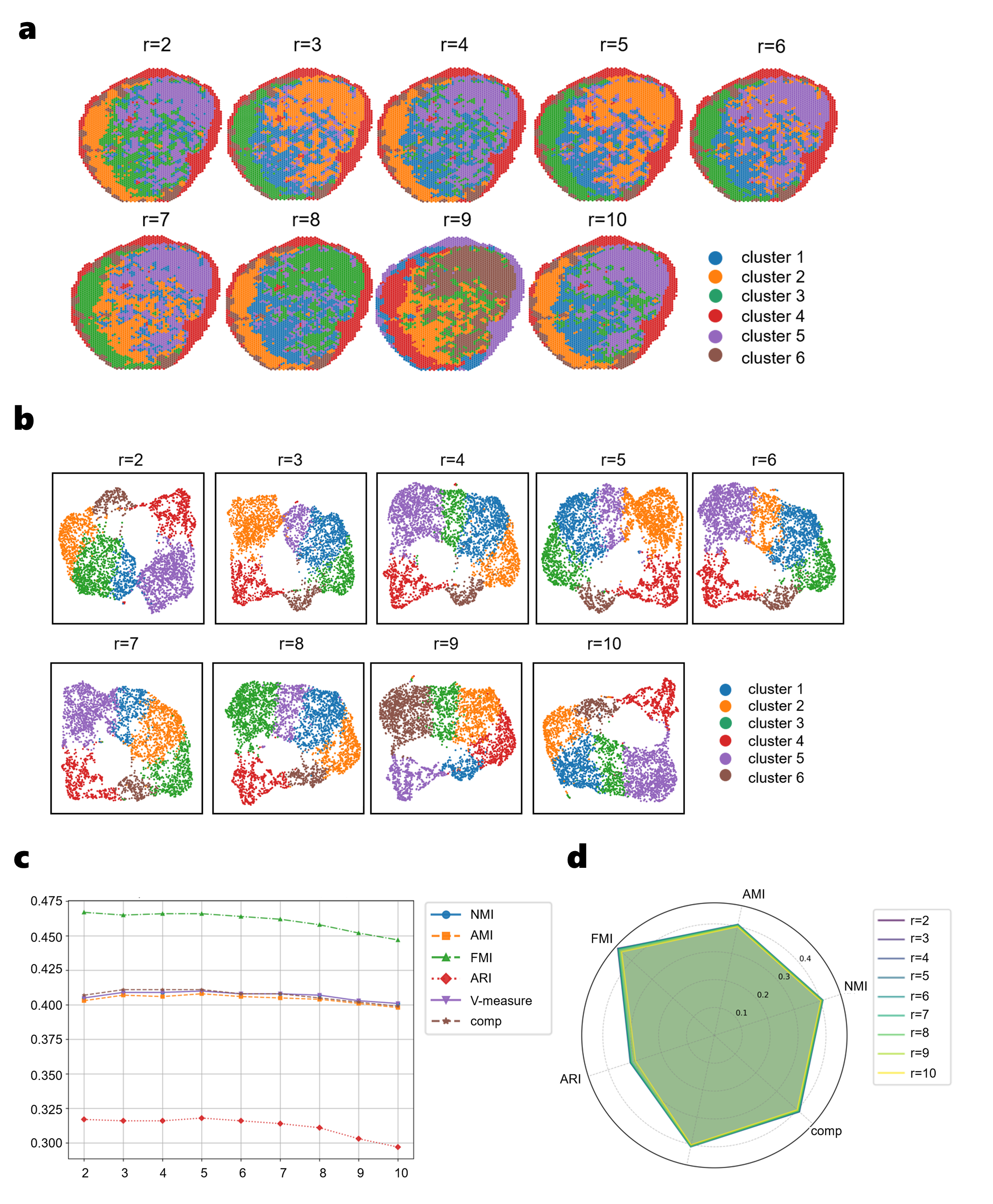


**Extended Fig. 7 | Performance of SpaBalance under Varying Spatial Neighbor Number (r) with Fixed Feature Neighbor Number (k=20) in the Human Lymph Node A1 Dataset.** a, Spatial plots with r=2, 3, 4, 5, 6, 7, 8, 9, 10. b, Clustering results. c, Line plots of clustering metrics. d, Radar plots of clustering metrics.

**Table S1. Hyperparameters of SpaBalance**

| **Parameter** | **Value** | **Notes / Usage** |
| --- | --- | --- |
| Learning rate | 0.0001 | Fixed for all datasets, Optimizer step size |
| β | 0.5 | Balancing factor for cross-modality consistency loss |
| λ₁ | 120 (SPOTS), 30 (10x Genomics Visium), 60 (Stereo-CITE-seq), 50 (spatial epigenome-transcriptome) | Dataset-specific weighting factor |
| λ₂, γ₁, γ₂, γ₄ | 1 | Weights for reconstruction and other auxiliary losses |
| γ₃ | 0.005 | Weight for cross-modality contrastive loss |

Default parameter settings demonstrate robust performance across the majority of datasets, but users are allowed to adjust them flexibly according to specific data characteristics and analysis tasks.

**Table S2. Parameter settings of baseline methods**

| **Method** | **HVGs / Features** | **Dimensionality reduction** | **Training setup** | **Notes** |
| --- | --- | --- | --- | --- |
| **Seurat WNN** | 2000 HVGs (RNA–protein), 3000 HVGs (RNA–ATAC/histone) | 30 (RNA), 18 (protein)10 (RNA & ATAC/histone) | Default | Used as preprocessing baseline |
| **totalVI** | Top 4000 HVGs | Automatic | scVI v1.2.2 | Designed for CITE-seq |
| **MultiVI** | Genes/peaks with >1% expression | 10 (RNA & ATAC/histone) | scVI v1.2.2 | Applied to RNA–epigenome data |
| **MOFA+** | All selected features | 15 latent factors | 500 iterations, default likelihoods (Gaussian for RNA, Poisson for counts) | Used official implementation |
| **SpatialGlue** | Default features | Default (per paper) | Default (per paper) | Used official settings |
| **PRAGA** | Default features | Default | Default (per code) | Used official implementation |
| **STAGATE** | Default features | Default | Default (per tutorial) | Originally designed for unimodal ST |
